# Towards understanding policy design through text-as-data approaches: The policy design annotations (POLIANNA) dataset

**DOI:** 10.1038/s41597-023-02801-z

**Published:** 2023-12-13

**Authors:** Sebastian Sewerin, Lynn H. Kaack, Joel Küttel, Fride Sigurdsson, Onerva Martikainen, Alisha Esshaki, Fabian Hafner

**Affiliations:** 1 Institute for Environment and Sustainability (IES), Lee Kuan Yew School of Public Policy, National University of Singapore, Singapore, Singapore; 2 Hertie School, Berlin, Germany; 3 Energy and Technology Policy Group, Department of Humanities, Social and Political Sciences, ETH Zurich, Zurich, Switzerland; 4 Institute of Science, Technology, and Policy, ETH Zurich, Zurich, Switzerland; 5 Climate Physics Group, Department of Environmental Systems Science, ETH Zurich, Zurich, Switzerland

**Keywords:** Climate-change policy, Computational science, Energy policy, Government, Law

## Abstract

Despite the importance of ambitious policy action for addressing climate change, large and systematic assessments of public policies and their design are lacking as analysing text manually is labour-intensive and costly. POLIANNA is a dataset of policy texts from the European Union (EU) that are annotated based on theoretical concepts of policy design, which can be used to develop supervised machine learning approaches for scaling policy analysis. The dataset consists of 20,577 annotated spans, drawn from 18 EU climate change mitigation and renewable energy policies. We developed a novel coding scheme translating existing taxonomies of policy design elements to a method for annotating text spans that consist of one or several words. Here, we provide the coding scheme, a description of the annotated corpus, and an analysis of inter-annotator agreement, and discuss potential applications. As understanding policy texts is still difficult for current text-processing algorithms, we envision this database to be used for building tools that help with manual coding of policy texts by automatically proposing paragraphs containing relevant information.

## Background & Summary

Climate change mitigation is one of the biggest challenges of our times. Yet, despite decades of climate action, public policy ambition remains insufficient, fuelling concerns that the commitment of the 2015 Paris Agreement to limit global warming to 1.5 °C is increasingly difficult to reach. Under this landmark agreement, the focus for policy action shifted from the international to the national level with the hope that governments would, over time, develop and adopt more effective policies across a wide range of sectors driven by their economic self-interest^1,2^. There is a broad consensus that existing policy action is not ambitious enough to reach the Paris goals^3,4^, however, there is notable uncertainty around how to measure the effectiveness of this policy action in high-profile analyses. For example, some studies build their analyses on policy ‘density’ (i.e., the number of policies)^5,6^, while others focus on targets or goals set by policies^7^. In particular, there is a substantial disconnect between these basic approaches that rely on easily accessible information, and the public policy literature where the focus is on policies’ specific ‘design’ rather than their density as driver of effectiveness^8^. Comprehensive data on climate policy design and its change over time remains very limited to date. Crucially, the causal link between the adoption of specific policy designs (policy output) and their impact on climate change mitigation (policy outcome) cannot be sufficiently established^9,10^. These data limitations therefore impede moving from ex-post analysis to ex-ante recommendations, which is central for public policy to have greater mitigation impact^11–15^.

Over the last decade, public policy literature has identified relevant ‘policy design elements’ that determine policy effectiveness^9,16–18^. These design elements span three levels of abstraction: ‘policy instrument types’ imply a high-level intervention logic (e.g. ‘regulation’ to allow or prohibit certain actions), ‘general policy design characteristics’ include basic design choices each policy comprises (e.g. its objective or addressee), and ‘specific policy design characteristics’ comprise design choices more specific to a certain policy field. Such a multi-level approach for conceptualising policy design elements draws on important insights from the literature: (1) The choice of policy instrument type is important, yet there is a growing consensus that no instrument type in climate change mitigation is superior to all others, rather a combination of different instrument types is needed to address a multitude of market failures and barriers^19–21^. (2) Any policy comprises a set of general design characteristics or elements irrespective of policy instrument types. For example, Cashore and Howlett^22^ conceptualise goals and means at different levels of abstraction and Schaffrin *et al*.^16^ propose six general design elements, namely objectives, scope, integration, budget, implementation, and monitoring. Importantly, for policy effectiveness, such general design characteristics are at least as important as the choice of instrument type^23,24^. (3) Regarding the socio-technical transitions necessary for decarbonising global economies, it has been shown that policy design that is ‘technology and/or application specific’, i.e., recognises different technologies’ need for custom support and/or is cognisant about different usages of technologies, is more effective in stimulating the adoption of carbon-neutral technologies^18,25–28^. Importantly, both general design characteristics and technology specificity have been shown to impact policies’ (dis-)continuation and ratcheting-up (-down) of ambition^29^. While efforts have been made to gather climate policy-related data, existing databases like LSE’s Climate Change Laws of the World^30^ and IEA’s Policies and Measures^31^ focus on general information (i.e., scope, topics, sectors covered, instrument types) rather than more specific assessments of policies’ design elements. This gap in existing datasets originates from a key challenge related to such data-gathering efforts: Data collection relies on manual coding conducted by trained staff, often in collaboration with country experts or government officials. This work-intensive approach is very hard to scale up, not least because funding for social science research on climate change issues is scarce^32^. As the ratcheting-up of climate policy ambition should be informed by the best available data, the lack of data on most of the policy design elements limits the systematic assessment of climate policies’ ambition and effectiveness. Therefore, alternatives to the manual coding of policies are urgently needed.

Text-as-data approaches leveraging machine learning and other analytic approaches can help scale up data-gathering in order to produce large and comparable datasets of policy design elements. Their ability to explore large amounts of texts has been gauged in the social sciences for a number of years^33–35^, with many applications focusing on party manifestos^36^ rather than public policy texts. Studies that do analyse public policies focus on individual design aspects (such as discretion given to bureaucracies^37^) and, most importantly, applications for generating data about climate policies are only emerging. For example, recent studies have applied text-as-data approaches to identify individual aspects of policy design, such as their topic or scope^38,39^ (e.g. adaptation to climate change^40^). In related fields like forest and environmental policy, researchers have used text-as-data approaches for identifying the tools (i.e., instrument types) used and objectives formulated^41,42^. Further approaches for assessing *general* and *specific* policy design elements at scale have, however, not yet emerged. In the absence of such approaches, analyses of policy effectiveness continue to rely on manually coded data^18,43–45^. Here, we provide a stepping stone towards the creation of large-scale climate policy datasets with text-as-data approaches. We believe that at this crossroads of methods development, policy scholars and data scientists should aim high. We transcend the prevailing focus on isolated design elements and develop an approach for identifying policy design elements in text more comprehensively.

To reach the long-term goal of (semi-)automatising the assessment of climate policy design elements to aid or replace resource-intensive hand-coding, this paper presents a training dataset that can serve to develop new analytical tools. The dataset substantially expands the scope of text-as-data approaches in policy analysis as it includes an encompassing set of policy design elements at different levels of abstraction. To achieve this, we developed a coding scheme that reflects these design elements and, crucially, defines machine-learnable tasks (see methods). To create the policy design annotations (POLIANNA) dataset, we selected a corpus of relevant EU climate policies (i.e., directives and regulations pertinent to climate change mitigation and renewable energy), developed a scheme for annotating instrument types, policy design characteristics, and technology and application specificity (for brevity, from here on simply technology specificity) at the level of text spans. We decided to annotate at a textually granular level both because it is closer to how human annotators analyse a policy, and to provide more flexibility for the type of analytical tools to be developed. We then manually coded a large body of text with a team of trained annotators. We extensively analysed the inter-annotator agreement in this complex setting, adapting a chance-corrected score and developing other agreement measures based on the F_1_ score (see technical validation). The resulting POLIANNA training dataset includes 18 policies containing 412 articles, i.e., subsections dividing the EU legal acts, comprising 20,577 annotated spans.

## Methods

The POLIANNA dataset is intended to support the efforts of policy scholars in analysing the ambitiousness and effectiveness of climate policy action at various levels^3,46–49^. The intended future identification tools to be developed on its basis would enable tackling many of the larger questions concerning the much-needed ratcheting up of climate policy ambition, such as policy effectiveness, the potential interplay between policies, and pathways for improving policy design over time. This is highly relevant for developing evidence-based policy recommendations. Specifically, we envision this dataset to be used, for example, to create a tool that helps with manual coding of policy texts by automatically proposing paragraphs that contain relevant information about policy design. In addition, the dataset can support the creation of tools to automatically extract specific information about policy design, such as, for example, the type of actor targeted. Some policy design information is context- and domain-specific and, therefore, likely still difficult to fully automatise. Consequently, we annotated the text at the most granular level in order to allow for the most flexibility in developing such tools.

**Fig. 1:**
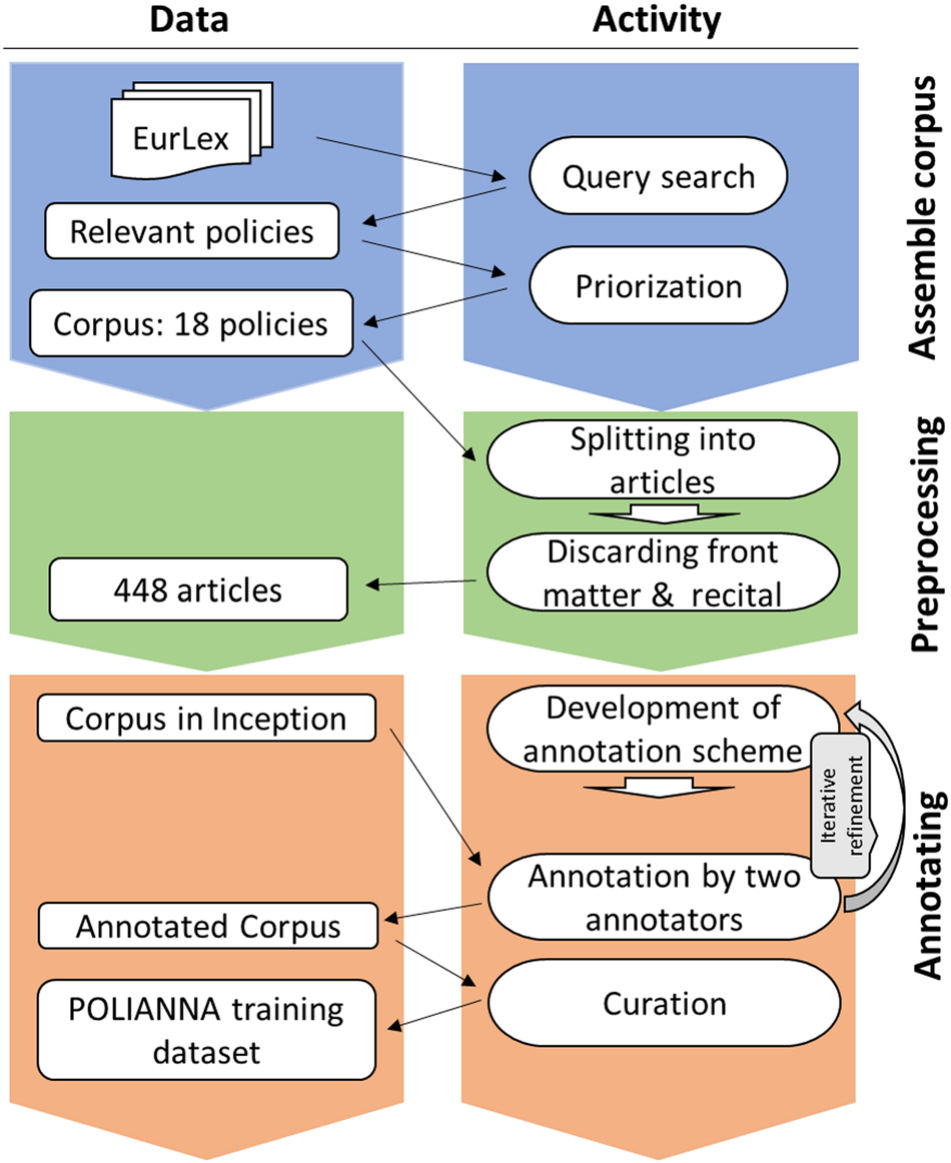
Work flow for creating the POLIANNA dataset. The activities are listed on the right, while different stages of the evolvement of the dataset are shown on the left.

To build this dataset, we have taken existing coding schemes that were designed for gathering information manually at the document level (i.e., complete policy texts)^16,18,50,51^ and adapted them to annotate text spans. Thus, we created a new scheme that fits statistical learning approaches and common tasks from the field of natural language processing (NLP). We then (1) assembled a corpus of EU policies, i.e., directives and regulations pertinent to climate change and renewable energy, (2) preprocessed the texts, and (3) extensively tested and revised the coding scheme, annotated a large body of text with a number of trained annotators (or “coders”), and curated the annotations for the final dataset (Fig. 1). In the following, we describe the three main steps of building the database in more detail, also highlighting methodological contributions in our work around developing and refining the coding scheme.

### Assembling the corpus

#### Policy selection

We sourced policy texts from EUR-Lex, the official website for accessing legal documents of the European Union. The website provides the texts to all EU legal acts open-source - i.e., regulations, directives, decisions, recommendations and opinions, which, for the purpose of our study we consider as constituting EU ‘policy’ - and comprises an ‘expert search function’^52^ that allows to run a query for identifying documents of interest. In an iterative process, we developed search queries that were intentionally broad in order to capture policies concerned with climate change mitigation in general as well as acts concerned with specific mitigation actions (i.e., different technologies to decarbonise relevant sectors). The search queries used can be found in the online appendix, together with the selection of metadata that we downloaded. Also note that, by default, EUR-Lex performs a full text search and searches for all words derived from the basic root word entered. EUR-Lex’s ‘expert search function’ offers two NEAR operators, NEAR10 and NEAR40. We opted to use the former, as many combinations of terms including “renewable energy” and/or “climate change” are possible and we wanted to initially capture a broad set of policies that we then narrowed down manually in a further step.

From the first broad catalogue of policies found through these queries, we selected a smaller corpus of policy texts for annotation: First, we sorted the texts by the percentage of articles that contain a set of energy and climate keywords. We adapted this set of keywords from the original query, refined them, and identified them using regular expressions. Second, we selected the top ten directives and regulations while excluding decisions due to their limited scope: Regulations have to be applied in their entirety across the whole EU, directives set a goal that EU Member States have to comply with by means of their own choosing, and decisions are rulings on specific cases by EU institutions that are binding only to those addressed. In the metadata, unique identifiers retain the information whether a policy text belongs to the category of regulations or directives. Third, we manually selected eight additional policies from those with high relevance based on their titles and keywords to fulfil additional requirements on the corpus. For example, we aimed to cover a variety of mitigation technologies and economic sectors, and included policies that are concerned more specifically with energy storage (i.e., battery storage) technologies and the transport sector (i.e., decarbonisation of transport through the deployment of electric vehicles) as, for instance, in Directive 2006/66/EC and Regulation (EC) No 79/2009, respectively. We also added landmark policies, such as one on electricity markets (Directive (EU) 2019/944). Following this rationale, we arrived at a selection of 18 policy texts representative of the EU’s overall policy approach regarding climate change mitigation and renewable energy technologies more specifically. The list of policies included can be found in the online appendix.

#### Preprocessing

As some of the policy texts are very long, we pre-processed the documents into shorter textual entities, namely the articles as specified in EU legal documents. This made the textual entities more comparable as they are more similar in length, and it helped assigning them to annotators. We annotated each article of each policy, besides the preamble (comprising citations and recitals), which we excluded from the labeled corpus as a policy’s legal basis or procedural information was not in our scope of interest. We made the script for dividing EU policies into articles available online. We split the 18 policies in 448 articles, and reduced those to 412 by excluding citations and recitals.

### Annotating

#### Coding scheme

We introduce a comprehensive coding scheme to capture three groups of policy design elements: instrument types, policy design characteristics, and technology specificity. The coding scheme is designed for annotation at the level of spans of one or more words (tokens), to allow annotators to highlight the exact text passage that was relevant to decide on a particular feature. For that, we had to considerably adapt existing coding schemes^16,18,50^ as these were designed to be implemented and compiled at the document-level only, resulting in the loss of information where design elements are occurring in the policy text. The authors of this study developed the coding scheme in an iterative process during annotator training.

For the annotations we used an open source labelling tool provided by TU Darmstadt, Inception^53^. The tool met all requirements for the annotation task, including allowing for annotations at the span-level, overlapping annotations, multiple annotators, and a hierarchical label structure. Inception enabled us to define a three-level hierarchical structure. On the highest level, we define three ‘layers’ that correspond to the three policy design elements introduced above, namely instrument types, policy design characteristics, and technology specificity. Within each layer, we define several ‘features’ that are to be identified in a text. For each feature, annotators could select from a set of ‘tags’ to annotate a span.

We experimented with different setups in a trial period, and then moved to refine the most workable scheme. This refinement involved finding the taxonomy and definitions of features and tags that would reflect relevant policy design concepts and at the same time would be applicable by annotators. After having arrived at a final coding scheme, we locked in the features and tags, and continued to iteratively refine a rule book accompanying the coding scheme in regular meetings. We also defined rules for annotating that would help with computerized information extraction regarding how to take into account contextual information, how much text to include in a span, and how to deal with overlap. The coding scheme is shown in Table 1 (see the online appendix for descriptions of the tags). The rule book that accompanies this coding scheme contains more detailed definitions, illustrative examples for each of the features, and discusses representative edge cases that coders have come across (see online appendix). A limitation of this approach is that we have not tested the coding scheme on inexperienced annotators after finalizing the rule book, however, one annotator who was brought in later in the process was able to understand and apply the scheme.

**Table 1:** Coding scheme developed and employed to create this dataset.

Layer	Feature	Tags
Instrument types	Instrument type	Voluntary agreement [VoluntaryAgrmt]
		Framework policy [FrameworkPolicy]
		Tradable permit [TradablePermit]
		Regulatory instrument [RegulatoryInstr]
		Tax incentives [TaxIncentives]
		Subsidies and direct incentives [Subsidies_Incentives]
		Research, Development & Demonstration (RD&D) [RD_D]
		Public Investment [PublicInvt]
		Education and outreach [Edu_Outreach]
		Unspecified [Unspecified]
Policy design characteristics	Actor	Default authority [Authority_default]
		Legislative authority [Authority_legislative]
		Newly established authority [Authority_established]
		Monitoring authority [Authority_monitoring]
		Default addressee [Addressee_default]
		Resources addressee [Addressee_resource]
		Monitored addressee [Addressee_monitored]
		Sector addressee* [Addressee_sector]
	Compliance	Sanctioning form [Form_sanctioning]
		Monitoring form [Form_monitoring]
	Reference to other policies	Reference to other policy [Ref_OtherPolicy]
		Amendment of policy [Ref_PolicyAmended]
		Reference to strategy or agreement [Ref_Strategy_Agreement]
	Objective	Quantitative target* [Objective_QuantTarget]
		Qualitative intention* [Objective_QualIntention]
		Quantitative target not mitigation [Objective_QuantTarget_noCCM]
		Qualitative intention not mitigation [Objective_QualIntention_noCCM]
	Resource	Monetary spending [Resource_MonSpending]
		Monetary revenues [Resource_MonRevenues]
		Other resource type [Resource_Other]
	Reversibility	Provision for reversibility [Reversibility_policy]]
	Time	Policy duration time [Time_PolDuration]
		Monitoring time [Time_Monitoring]
		Resources time [Time_Resources]
		Compliance time [Time_Compliance]
		In-effect time [Time_InEffect]
Technology specificity	Technology specificity	Low-carbon technology* [Tech_LowCarbon]
		Other technology [Tech_Other]
	Energy specificity	Low-carbon energy source or carrier* [Energy_LowCarbon]
		Other energy source or carrier* [Energy_Other]
	Application specificity	Low-carbon application* [App_LowCarbon]
		Other application [App_Other]

Asterisked tags are specific to climate change mitigation and renewable energies and thus are policy-field specific. For further information on features and tags see the online appendix.

We chose to label at the level of spans in order to offer maximum flexibility for future prediction tools that can be developed with these granular data. Labels at the span level can easily be aggregated to sentence, paragraph, or document levels. Given the high amount of text comprehension and domain knowledge that is needed for processing and classifying some of the policy design elements, we expect that for many elements only semi-automated tools with a human in the loop will achieve satisfactory performance. Annotating at the span level allows for training a model that can highlight passages in the text relevant for a certain element, and provide interpretable information for humans supervising the annotation. Potential NLP tasks on these annotated spans are named entity recognition, sentence classification (if simplified to the sentence level), or document classification (if simplified to the paragraph level). Moreover, in principle every tag can be treated as a separate task, but tags can also be aggregated if the research design requires it. Our hierarchical structure naturally allows for such aggregation.

There are some particularities of our coding scheme that should be noted: First, the main objective of our work on policy design is to analyse climate change mitigation and renewable energy policies. However, the underlying logic of the coding scheme and many of its features are generalisable and thus apply to policies in other areas as well. The features created by us, therefore, can be distinguished between those particular to the case of climate change mitigation and renewable energy technologies, and those that are general (as noted in Table 1). Second, while most tags are mutually exclusive, the actor tag set is not designed to be so. This set of tags has an implicit fourth hierarchical level, as the tags are divided in “addressee” and “authority,” - thus speaking to the important question of which actors are the recipient of policy action and which are responsible for it - and then contain further subcategories. As these subcategories can be difficult to code, it was most operational to create a “default” category that can be applied if the more specific ones cannot. For analyses where the subcategories are irrelevant, we recommend aggregation to the “addressee” and “authority” categories.

#### Annotation and curation

We annotated at the level of spans, which consist of one or more tokens. Annotators were both required to specify the boundaries of the relevant span (unitising) and the correct label of that span (categorisation). Furthermore, we enabled the following annotation options based on the taxonomy introduced by Mathet *et al*.^54^: Embedding (hierarchical overlap), free overlap, and sporadicity. We did not specify the length of spans a priori but instructed the annotators to label the shortest acceptable span for a given instance to be meaningful.

The data were annotated by four authors with relevant graduate-level expertise in political science and renewable energy technology and policy. The annotators received extensive training in several dozen meetings and conducted several trial runs leading up to the development of the final coding scheme, during which all edge cases they encountered were discussed. The annotation task generally required a high level of subject-matter expertise. The whole corpus was annotated by two annotators and then curated by one of the corresponding authors of this paper or, in few cases, one of the annotators supervised by the corresponding authors. The curator aggregated the labels from both annotators and resolved conflicts, as well as added new labels and dismissed those that appeared false, if necessary.

Overall, the annotators spent more than 600 hours in total to create the dataset, which - considering duplicate annotations and the development of the coding scheme - would mean that around a hundred hours are required for annotating 18 policies (or 412 articles) by hand with the full coding scheme. This means that annotating a single policy of a typical length of 30 articles would require more than six hours. As empirical (*large-n studies*) analyses of policies’ effectiveness would require design data of, ideally, several hundred policies, the cost for manual annotation would be prohibitively high.

### Resulting dataset

**Fig. 2:**
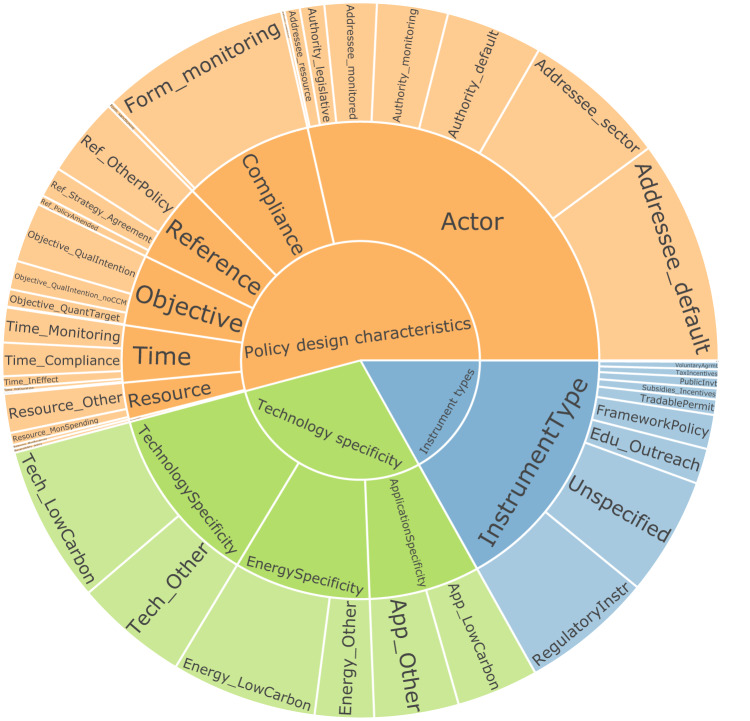
Distribution of annotations in the dataset. The circles correspond to layers, features, and tags (from the inside out). The colors highlight the three layers we annotated: Blue comprises layers, features and tags belonging to instrument types, orange those belonging to policy design characteristics, and green those belonging to technology specificity. The wider a wedge, the larger is the share of annotations belonging to this particular layer, feature, or tag.

We summarize the number of annotations per layer, feature, and tag in Fig. 2. More than half of the annotations in the dataset are policy design characteristics, and instrument types make up less than a quarter of the annotations. While for technology specificity the annotations are relatively uniformly distributed for the respective features and tags, this is not the case for the other layers, where we see that the annotations are dominated by some tags, while a large number of tags occur less frequently. The tag that appears the least frequent overall is “quantitative target not mitigation” (Objective_QuantTarget_noCCM) with 8 annotations, while “default addressee” (Addressee_default) occurred the most with 2079 annotations. Some patterns we observe in the distribution between annotations can be expected from what we know about policy design. For example, we should see less instrument types mentioned in the text than tags of the other layers, as that terminology is rather abstract and, once the instrumental logic is established in a policy, the focus of the design should move to more specific characteristics that determine how an instrument is designed.

Policy design characteristics are dominated by the actor features and addressee tags (especially Addressee_default and Addressee_sector). This is a particularity of our coding scheme, where we coded all actors as addressees that are subject to policy actions, even when this means that they might act as an authority in other contexts (see rule book in the online appendix). As EU directives specify what Member States ought to do, all mentions of Member States in this context are coded as addressees. The second-smallest number of annotations are attributed to the “resource” feature, which encompasses the allocation of monetary, institutional, and other resources to achieve a policy goal. Given how important the allocation of financial resources is for the success of policies^55^, this marginal focus comes as a surprise. Taking a closer look at the instrument type layer, we see that RegulatoryInstr tags appear most frequently, which is to be expected given that the EU places a large focus on regulation in its policy-making. Another substantial share of annotations belong to the category of Unspecified policy instrument type. This is driven by references to general policy action to be taken up by Member States.

**Fig. 3:**
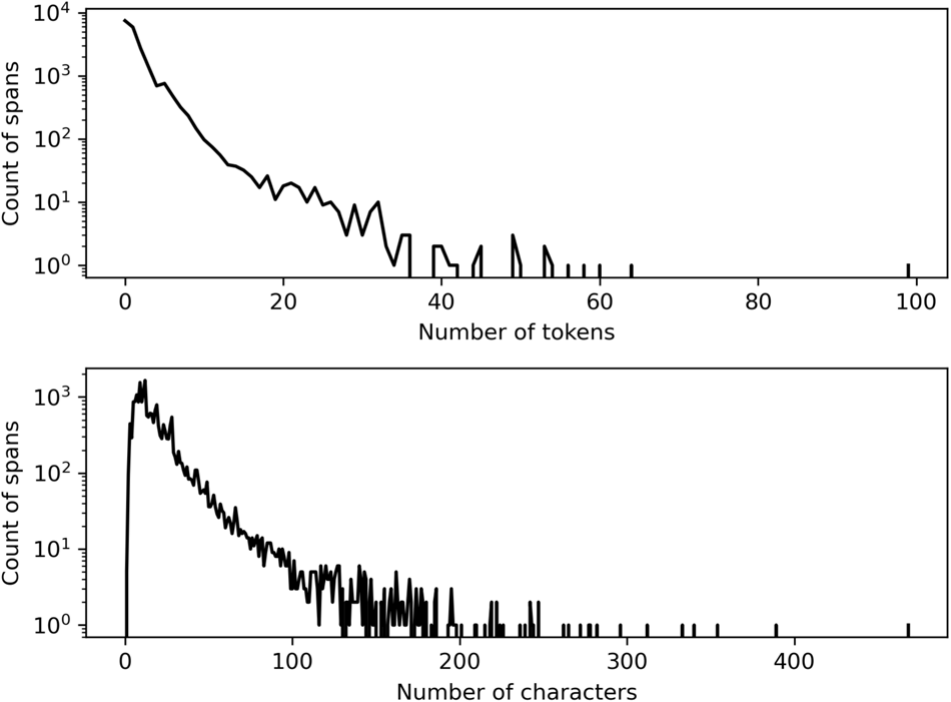
The distribution of the length of the annotated spans in the curated dataset, as measured in the number of tokens and characters per span.

We find that the length of annotated spans follows a very skewed distribution, with most spans being short and few that are very long (Fig. 3). Examining the corpus reveals that the distribution of span lengths differs between tags, some tags generally contain longer annotations (for example those describing “objectives”) while others typically contain very short annotations (for example those describing “actors”). The longest annotated span is a description of how to reverse a particular act, annotated as “provision for reversibility”, which still only encompasses a part of one sentence. More than 77% of spans have 3 or less tokens (i.e. they are 1-, 2-, or 3-grams). This means that for developing ML models named entity recognition and sentence classification are promising approaches for predicting most of the tags.

## Data Records

The dataset can be accessed on Zenodo^56^ at https://zenodo.org/records/8284380. The annotated corpus contains 18 policies, which are split in 412 documents (‘articles’) that correspond to an article in each of the policies. Those splits have no other meaning but to structure the data as the focus for training a machine learning approach should be on the (sub-)sentence level. Crucially, the annotation at the (sub-)sentence level includes 20,577 annotated spans containing 52,298 tokens. The policy texts are obtained from EUR-Lex^52^. Metadata is provided in the folder “01_policy_info”, which contains the full coding scheme in JSON format and a CSV file with metadata about each policy. Metadata is directly extracted from EUR-Lex^52^ and includes the policy’s ELI, CELEX number, title, and other relevant data about dates and policy fields.

The data can be downloaded in three different formats: The folder “02_processed_to_dataframe” contains the data in pickle format as a Python object. This is the recommended method when using our own Python framework to work with the data, which is provided on the GitHub project page https://github.com/kueddelmaier/POLIANNA. The folder also provides the data as a CSV file. The remaining folders contain the dataset in different JSON formats. A human-readable format would be available in “03b_processed_to_json.” In this folder, each article corresponds to a sub-folder, where the curated annotated spans are stored in a separate JSON file ("Curated_Annotations.json"). Every line of the dataset corresponds to one span, and the data contain the span ID, layer, feature and tag of the annotation, start and stop character, the annotated text and the tokens contained in the span. Each sub-folder also provides the data in form of annotated tokens ("Tokens.json"), and further contains the raw text of the article as a text file ("Raw_Text.txt") and metadata about the policy ("Policy_Info.json"). The file ("Coder_Annotations.json") contains the annotations of the two annotators (before curation). The folder "03a_processed_to_jsonl" has the overall structure as the JSON format, but the spans and tokens are stored in a JSONL format (newline-delimited JSON).

## Technical Validation

Here, we present inter-annotator agreement (IAA) results for the annotated dataset. Because of the rather complex annotation setup, we present the results for several IAA measures, including one that we adapted from the *γ* indicator by Mathet *et al*.^54^. Each policy in our dataset was independently annotated twice, which allows us to compute IAA scores for the whole dataset. Additionally, we also consider the agreement between annotators and curators.

### Inter-annotator agreement

**Fig. 4:**
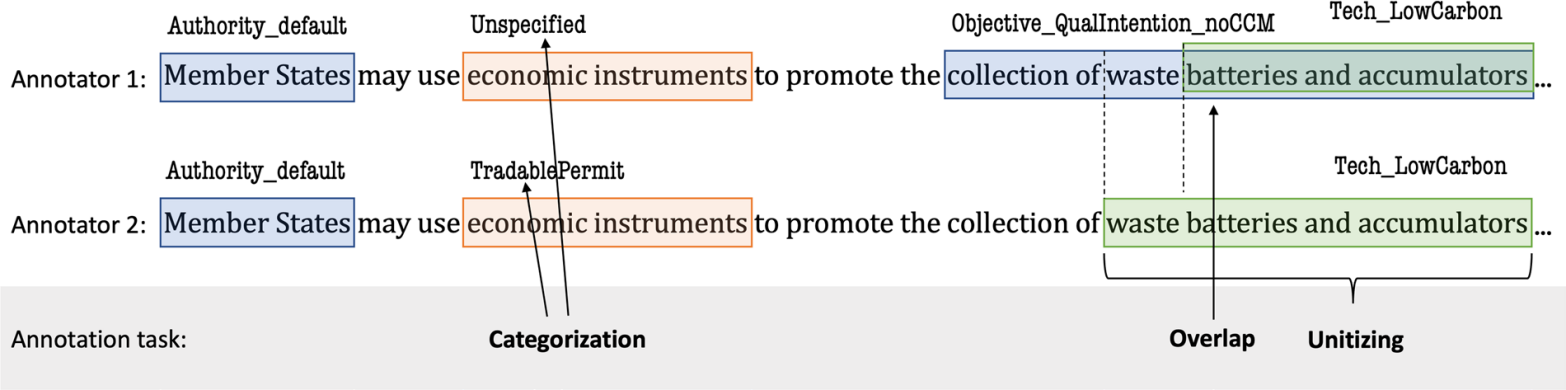
llustration of categorisation, unitising, and overlap using an example sentence from the policy EU_32006L0066. Adapted from Mathet *et al*.^54^.

The inter-annotator agreement is a measure of agreement between annotators in computational linguistics and text-as-data approaches^57^. Various terminology such as inter-coder reliability, inter-coder agreement, inter-rater reliability, or similar, is used for the measure. The IAA allows to evaluate the reliability between different annotators, i.e. to what extent they agree on the same annotation task. If annotators are not consistent, they may lack the necessary coding skills or the task and coding scheme are not clearly defined. The state of the art IAA measures are computed in relation to the agreement expected by chance (chance-corrected). IAA measures can moreover serve as a benchmark for evaluating automated annotation approaches, such as ML models. The performance of ML models is typically not evaluated with chance-corrected scores, which is why here we use both chance-corrected IAA scores and performance measures common in ML. Moreover, due to the sparsity of annotations and large number of classes, agreement by chance is less likely in our case. Our complex labelling task requires annotators both to choose the location and length of the span (unitisation) and choose the tags (categorisation) (Fig. 4). Moreover, we allow overlapping annotations. This means that standard measures, such as Cohen’s *k* or Krippendorff’s *α*, cannot be applied here^54^. Instead, we use a number of alternative approaches to measure the agreement in our dataset. Evaluating unitisation and categorisation requires both to match annotations between annotators to account for unitisation and overlap, as well as to measure the agreement of the categories. A unified and chance-corrected measure for IAA with unitisation and categorisation is provided with the *γ*-score^54^. We adopted the *γ*-score for evaluating the chance-corrected IAA, and we also provide simple agreement measures that are not chance-corrected and suitable as benchmarks for ML model evaluation. We base these on the F_1_ score, which is the harmonic mean of precision and recall, where precision measures the ratio of true positives of all predicted positives, and recall measures the ratio of correctly predicted positives to all true positives

1 $${F}_{1}=2\frac{{\rm{precision}}\cdot {\rm{recall}}}{{\rm{precision}}+{\rm{recall}}}=\frac{tp}{tp+\frac{1}{2}\left(fp+fn\right)}.$$

We compute the F_1_ score between two annotators by defining one of the annotators as the ground truth and the other annotator as the prediction. As the F_1_ score is not symmetric between annotators, we do this twice with each annotator as the ground truth, and then average over both. For comparison with the curation, we use the curation as the ground truth and average over both annotators. Here, we describe the different IAA scoring functions we used, and provide a summary and results in Table 2.

**Table 2:** The different IAA measures and results, comparing the two annotations (ann.-ann.) and the annotations to the curation (ann.-cur.). Results are computed by article and then weighted and averaged by the number of tokens. For the annotations to the curation, results are averaged over the two annotators.

name	unitising match	categorisation match	chance correction	ann.-ann.	ann.-cur.
*γ*	unified approach		yes	0.50	0.63
*γ* soft penalty	unified approach		yes	0.55	0.66
F_1_ exact	exact span	exact	no	0.37	0.57
F_1_ heuristic	partial span (multi-way matching)	exact	no	0.49	0.64
F_1_ token-wise	exact token	exact	no	0.46	0.63

### Scoring functions

#### Gamma (*γ*)

The *γ* score is a chance-corrected measure that can handle unitising, categorisation, and overlap. It is *unified* as it measures agreement and aligns annotations at the same time. It was introduced by Mathet *et al*.^54^ to address the shortcomings of existing IAA measures for the more complex case of unitising with overlap, and can be used for different continuous data such as text or audio files. To align annotations, the *γ* algorithm computes a score (“disorder”) of all local disagreements between annotators, and finds the alignment of annotations that minimizes the overall disorder. This minimal disorder is then chance-corrected by using an “expected disorder” obtained by randomly re-sampling annotations. The *γ* score calculates the agreement both in terms of the positioning and the agreement in categories. By defining the categorical dissimilarity between tags, one can also soften the penalty for two tags depending on how similar they are. As a default, we give any disagreement between tags an equal penalty. We also implement a soft penalty by defining the distance between two tags within the same feature as 0.5, instead of 1 which is used for entirely dissimilar categories. The *γ* score ranges from 1 (perfect agreement) to 0 (no agreement). Refer to Mathet *et al*.^54^ for details, and Titeux and Riad^58^ for the Python implementation of the score, which we use in this study.

#### F_1_ exact

Annotations are aligned based on exact match, which means that they need to fully agree in both unitisation and categorisation. We count those exact matches as true positives when computing the F_1_ score, and all unmatched annotations as false positives and false negatives. This is a very strict interpretation of a match that also penalises the practice of annotating overlapping spans with the same tags, which we encouraged in our coding scheme in some cases. For example, one annotator may have correctly labeled the text “battery and storage technology” as well as “battery” and “storage technology” with the tag Tech_LowCarbon, resulting in hierarchical overlap. The second annotator may only have “battery and storage technology”, which is also acceptable, however, the F_1_ *exact* score would count false positives. To address this issue, we introduced the F_1_ *heuristic* score below.

#### F_1_ heuristic

For F_1_ *heuristic*, annotations between two annotators, or annotator and curation, are counted as an agreement if they agree in category and there is any overlap. Additionally, we do not require a one-to-one match anymore, which results in a more generous performance when evaluating the agreement. Using the example from above, both “battery and storage technology” as well as “battery” and “storage technology” can now be counted towards the same ground truth annotation. More generally, denoting the annotator by *A* and the ground truth (chosen either as the other annotator or the curator) by *GT*, we define for every tag:

*tp* = # labeled spans in *A* that have a categorisation match and overlap with any spans in *GT*,

allowing for matching multiple tags in *A* to the same tag in *GT*.

*fp* = # labeled spans in *A* that do not have an overlapping span in *GT* with a categorisation match.

*fn* = # labeled spans in *GT* that do not have an overlapping span in *A* with a categorisation match.

To implement this score, we only search for one counterpart with the same category, which at least partially agrees on the location of the span. The score naturally leads to an asymmetry between the scores for two annotators. For instance, the example of “battery and storage technology” might be annotated as one span by *A* and two spans by *GT* (only “battery” and “storage technology”). Here, we count *tp* = 1, and we would count *tp* = 2 if roles were reversed. We therefore average the scores of both annotators if we are comparing two annotators.

#### F_1_ token-wise

The score compares the number of annotated tokens that agree fully in categorisation (there is no unitisation here). This scoring function takes into account that in some cases it is more meaningful to measure how much of the overall text is annotated in the same way, and it gives more weight to longer spans that are in agreement. We compute the F_1_-score based on the number of agreeing tokens between two annotators or one annotator and the curation. For the case of annotator (*A*) and ground truth (*GT*), the score is computed as follows:

*tp* = # labeled tokens shared between *A* and *GT*,

*fp* = # labeled tokens in *A* that are not in *GT*,

*fn* = # labeled tokens in *GT* that are not in *A*.

For overlapping annotations, we apply the same rule as described for F_1_ heuristic.

### Scoring results

We compute the scores for all articles, which are each labeled by two annotators, and then average over all articles (weighted by the number of tokens in the articles). We also compare the performance of the annotators to the curation (averaged over both annotators and all articles). Refer to Table 2 for the results. The annotator-annotator IAA is not very high, which is not unexpected given how complex the annotation task was. Note that for all scores (besides the soft-penalty *γ*-score) we require exact agreement for the categories. Some tags within the same feature are rather difficult to distinguish from another (e.g. Addressee_default and Addressee_resource), which may be a reason that the soft-penalty *γ*-score is somewhat higher than the *γ*-score without soft penalty. The F_1_ *heuristic* score is considerably higher than F_1_ *exact*, which shows that allowing for flexibility in unitisation improves the agreement.

**Fig. 5:**
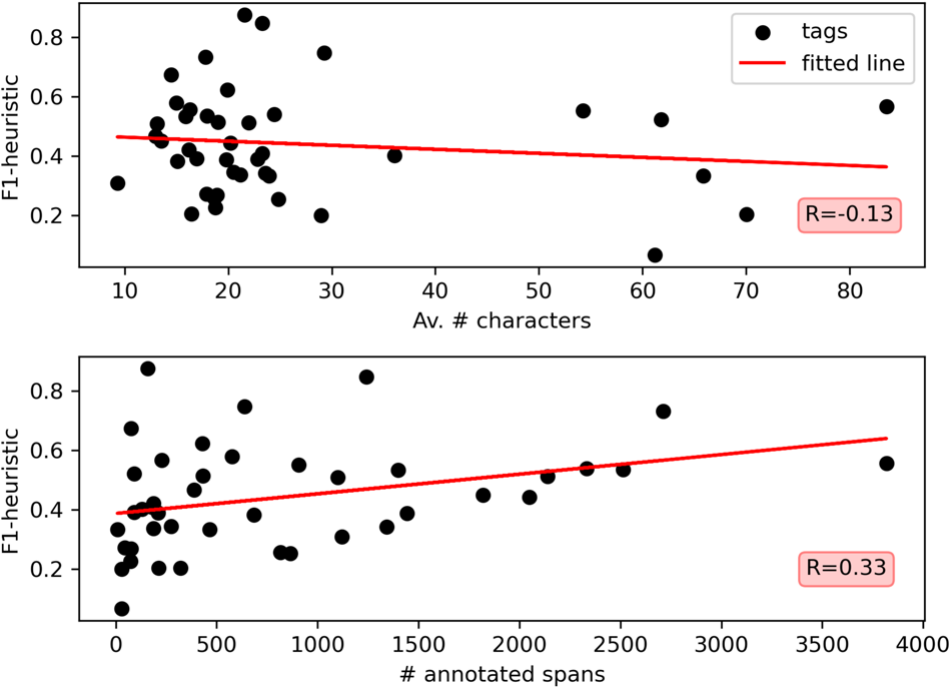
Inter-annotator agreement by tag depending on the average number of characters in the annotated spans (above) and the number of annotated spans with that tag (below). The Pearson correlation coefficients are given in the red box.

Another point of interest is how much the IAA differs between different features or tags. Comparing the performance with the F_1_ *heuristic* score (span-weighted), we find that the “resource” feature has the lowest agreement of 0.28 and “reference” the highest with 0.80. The good performance of reference annotations is expected, as there is clear formatting associated with references to other policies. Taking a closer look at the tags, we found even larger divergence in performance. The lowest score of a tag belongs to Reversibility_policy that rarely occurred in the dataset, and where the text passages are long in average and may be quite different from one another. The three tags that perform best belong to the “reference” feature, with Ref_PolicyAmended scoring at 0.88. Those occur frequently and exhibit distinct formatting. We tested if the length of the spans and the number of occurrences in the text relate to the performance but found only a small correlation with the number of annotations per tag (Fig. 5). We expect other factors that may make some tags easier to code than others, such as the semantic and lexical diversity between annotations. Further research is needed here.

We have also compared the performance of each of the four annotators to the curation. Those differ considerably, with the F_1_ *heuristic* score for the best performing annotator being 0.74 and the lowest being 0.45. This indicates that although all four annotators received the same extensive training, a great amount of skill and background knowledge was required to implement the coding scheme.

## Usage Notes

The annotated dataset can be used to build automated tools that help with assessing policy design elements at scale. Primarily, we envision this data to be used for developing learning-based models to identify text passages containing relevant information about policy design. Such tools can support much faster and detailed human annotation, and in certain circumstances may also allow for fully automated annotation of text. As such, this dataset provides a first stepping stone towards the automatic assessment of policy design elements that is needed for scaling up policy analysis in crucial fields like climate mitigation.

With this dataset, we provide various scripts to pre-process the annotations, run descriptive statistics, and compute the IAA. We also provide tools that may aid in extending the dataset by downloading and splitting the EU policies into paragraphs.

While the POLIANNA dataset includes only EU policies on climate change mitigation and renewable energy, the underlying approach is designed to be applicable to other legislative contexts and policy fields. We therefore expect many tags to generalise well to new policy fields. As highlighted in Table 1, we both annotated field-agnostic tags and renewable energy-specific tags that are less relevant in other policy fields. Also, applying the approach to policies’ preambles, which were not labelled in the POLIANNA dataset, would be possible and potentially of interest for legal scholars interested in policies’ legal basis and similar issues. While the coding scheme as such is applicable to other jurisdiction, it is an open question how well a model trained on EU texts would generalise to laws of other jurisdictions, and here creating additional training data may be necessary. The creation of further data for inference and training will be readily possible with the material provided here, the online appendix, and the code repository. For example, including annotated texts from the US legislative contexts would only require adapting the text preprocessing to the form in which US policy texts are available.

For building automated annotation tools, we suggest taking different approaches depending on the feature or tag. As noted above, the length, frequency, and similarity of annotated spans differ between features or tags. Some, such as the “actor” feature, occur frequently and are dominated by few short expressions (e.g. “member state”), while others, such as the tag Objective_QuantTarget, occur much more rarely and contain long and varied expressions, e.g. in the form of “union target of at least a 32% share of energy from renewable sources in the union’s gross final consumption of energy in 2030.” This means that different text as data methods are needed to extract different policy design features or tags from the documents. For many of the features or tags, sentence classification may be an appropriate approach for identifying relevant text passages. This approach would require the annotations to be extended to the sentence level. Particularly for the tags that are dominated by short expressions, named entity recognition may be an interesting approach for automatically annotating spans. Question answering may be another applicable task for some features and tags, and certain research questions. Based on inherent characteristics of annotated examples, as well as the IAA results, we expect the difficulty of automated approaches to vary greatly between tags and features. Moreover, those features or tags with only few examples in the dataset may be more difficult to learn than those with many data points. To increase the sample size, and avoid confusion between similar classes, it may help to aggregate certain tags, depending on the task at hand.

By developing tools for the automated assessment of policy design characteristics, the POLIANNA dataset enables public policy scholars to engage with a number of different research questions. Potentially interesting areas to explore include examining how policy design changes over time, how the design differs between different authors of the legislation or between different technologies and sectors addressed, and how it relates to actors’ behavior.

1. How does policy design change over time? Are there discernible trends towards or away from specific policy design elements, e.g. which actors are addressed or what targets are defined? Do such trends collectively amount to ‘major’, ‘paradigmatic’, or ‘transformative’ policy change as discussed in the public policy literature^22,59,60^? Given the magnitude of societal challenges that require policy intervention, such analyses would contribute greatly to the understanding of our societies’ capacity to tackle existential crises such as climate change. To analyse this research question, all tags are potentially relevant, and the presence of specific design elements would need to be tracked over time.
2. Does policy design depend on who is the designer? Are policies designed by (non-elected) bureaucrats or regulatory agencies different to policies designed by (elected) parliamentarians^61^? For the field of climate policy, the argument has been made that bureaucratic policy-making produces more ambitious and effective policies^62^. The training dataset already includes both EU regulations designed by bureaucrats and directives designed by legislators, and it provides a basis for investigating this claim empirically.
3. Does policy design change when targeting different (low-carbon) technologies? Are policy design choices influenced by differences in the inherent technological characteristics, such as technologies’ complexity^27^? Analysing whether distinct types of technologies (using the technology specificity) are targeted with specific policy design choices (e.g. the instrument types) would contribute to broader discussions about the governance of (emerging) technologies^63,64^. Such analysis can also help to identify what constitutes ‘technology-smart’ policy design that is successful in scaling the research, development and deployment of specific technologies^28^.
4. Does policy design differ across policy fields? Are similarities greater between related policy fields (like climate change mitigation and adaptation) than more distant policy fields (like climate change mitigation and social welfare)? Analysing policy design across fields would contribute to understanding whether countries develop discernible policy styles (e.g. reactive rather than anticipatory decision-making and a preference for top-down regulation) based on their unique cultural and institutional features^65^ or whether they are prone to innovate more in policy fields marked by increased levels of diffusion of design ideas (like renewable energy policy)^66^. For this, the approach would need to be used on a set of policies from different fields.
5. Does a detailed assessment of policy design allow modelling actors’ behaviour? If the assumption is that targeted actors will react to the (often very complex) set of rules that are expressed in each sentence of a policy text, their behaviour could be analysed and predicted. Research programmes interested in this link between rules and behaviour, like the *Institutional Grammar* school of thought^67^, so far struggle with scaling-up their micro-level assessments of such rules^68,69^ despite first advances to use ML approaches^70^. Our POLIANNA dataset similarly allows for analyses of how different actors are targeted by rules by relating them to further policy design elements, and could contribute to this line of research.

The POLIANNA dataset represents an important step towards leveraging ML approaches for public policy analysis. With this labelled text corpus covering an encompassing set of policy design elements, we offer many inroads for the development of analytical tools to speed up the hand-coding of policy design. The development of such tools is crucial for enhancing our understanding of policy design for tackling the grand societal challenges of our times like climate change.

### Supplementary information

Appendix & Datasheet

## Data Availability

Accompanying scripts are made available at https://github.com/kueddelmaier/POLIANNA. The repository contains scripts to split the raw policy text retrieved from EUR-Lex into articles, to process new data labeled with Inception, and to generate summary statistics.
