# Supplementary material for: Towards understanding policy design through text-as-data approaches: The policy design annotations (POLIANNA) dataset
Source: Sci Data. 2023 Dec 13;10:896. doi: 10.1038/s41597-023-02801-z (PMC10719256; doi:10.1038/s41597-023-02801-z)
Supplement: Supplementary file 1 — Appendix & Datasheet [file 41597_2023_2801_MOESM1_ESM.pdf]

Appendix

I. Search terms

As mentioned in the main text, we used the “expert search function” of EUR-Lex to identify laws of interest. The search queries below are intentionally broad in order to capture legal acts concerned with climate change mitigation in general as well as acts concerned with specific mitigation actions (i.e. different technologies to decarbonise relevant sectors). From the set of legal acts found with these search queries, we identified the 18 legal texts used for developing the POLIANNA dataset.

1. Renewables
   TE~ (renewable NEAR10 energy) OR (alternative NEAR10 energy) OR (low-carbon NEAR10 energy) OR (non-fossil NEAR10 energy) OR (sustainable NEAR10 energy) OR (clean NEAR10 energy) OR (green NEAR10 energy) OR (low-carbon NEAR10 energy) OR (renewable NEAR10 electricity) OR (alternative NEAR10 electricity) OR (low-carbon NEAR10 electricity) OR (energy NEAR10 efficiency) OR (energy NEAR10 innovation) OR (energy NEAR10 technology) OR (energy NEAR10 efficiency) OR (renewable NEAR10 resources) OR (decarbonisation) OR (energy NEAR10 act) AND DTS_SUBDOM = LEGISLATION AND FM ~("Regulation” OR "Directive" OR "Decision") AND AU_CODED = EP NOT FM_CODED = CORRIGENDUM AND DD >= 01/01/2000 <= 31/12/2019

2. Climate
   (TE~ (climate NEAR10 change) OR (climate NEAR10 commitment) OR (climate NEAR10 justice) OR (climate NEAR10 legislation) OR (greenhouse NEAR10 warming) OR (greenhouse NEAR10 gas) OR (greenhouse NEAR10 effect) OR (global NEAR10 warming) OR (carbon NEAR10 tax) OR (carbon NEAR10 footprint) OR eco-efficiency OR “kyoto protocol” OR methane OR “nitrous oxide” OR “sea level rise”) AND DTS_SUBDOM = LEGISLATION AND FM ~("Regulation” OR "Directive" OR "Decision") AND AU_CODED = EP NOT FM_CODED = CORRIGENDUM AND DD >= 01/01/2000 <= 31/12/2019

3. Hydro
   DTS_SUBDOM = LEGISLATION AND FM ~("Regulation” OR "Directive" OR "Decision") AND AU_CODED = EP NOT FM_CODED = CORRIGENDUM AND DD >= 01/01/2000 <= 31/12/2019 AND (TE ~("hydro-electric" OR hydroelectric OR (hydro NEAR10 dam) OR "water-power" OR waterpower OR hydropower OR (pumped NEAR10 hydro) OR (pumped NEAR10 storage) OR "hydro-energy" OR (hydro NEAR10 energy) OR (hydro NEAR10 generation) OR (hydro NEAR10 capacity) OR (hydro NEAR10 penetration) OR (hydro NEAR10 share) OR (hydro NEAR10 plant) OR (hydro NEAR10 project) OR ("small-scale" NEAR10 hydro)))

4. Wind
   DTS_SUBDOM = LEGISLATION AND FM ~("Regulation” OR "Directive" OR "Decision") AND AU_CODED = EP NOT FM_CODED = CORRIGENDUM AND DD >= 01/01/2000 <= 31/12/2019 AND (TE ~(wind NEAR10 energy) OR (wind NEAR10 power) OR (wind NEAR10 electricity) OR (wind NEAR10 generation) OR (wind NEAR10 capacity) OR (wind NEAR10 penetration) OR (wind NEAR10 share) OR (wind NEAR10 plant) OR (wind NEAR10 system) OR (offshore NEAR10 wind) OR (onshore NEAR10 wind) OR (wind NEAR10 turbine) OR (wind NEAR10 mill))

5. Solar
   DTS_SUBDOM = LEGISLATION AND FM ~("Regulation” OR "Directive" OR "Decision") AND AU_CODED = EP NOT FM_CODED = CORRIGENDUM AND DD >= 01/01/2000 <= 31/12/2019 AND

(TE ~((solar NEAR10 energy) OR (solar NEAR10 cell) OR (solar NEAR10 panel) OR photovoltaic* OR (solar NEAR10 PV) OR (PV NEAR10 system) OR (photovoltaic NEAR10 project) OR (solar NEAR10 power) OR (solar NEAR10 electricity) OR (solar NEAR10 generation) OR (solar NEAR10 capacity) OR (solar NEAR10 share) OR (solar NEAR10 plant) OR (open-field NEAR10 PV) OR (rooftop NEAR10 photovoltaic) OR (utility NEAR10 photovoltaic) OR (building-integrated NEAR10 photovoltaic) OR (solar NEAR10 collector) OR (solar NEAR10 heat) OR (solar NEAR10 cool) OR (solar NEAR10 light) OR (solar NEAR10 market) OR (solar NEAR10 project) OR (photovoltaic NEAR10 system) OR (PV NEAR10 array) OR (solar NEAR10 array) OR (photovoltaic NEAR10 cell) OR (PV NEAR10 cell)))

6. Fuell cell (hydrogen)
   DTS_SUBDOM = LEGISLATION AND FM ~("Regulation” OR "Directive" OR "Decision") AND AU_CODED = EP NOT FM_CODED = CORRIGENDUM AND DD >= 01/01/2000 <= 31/12/2019 AND (TE ~(hydrogen NEAR10 fuel) OR (hydrogen NEAR10 energy) OR (fuel NEAR10 cell) OR (biomass NEAR10 gasification) OR electrolysis OR electrofuel)

7. Distributed generation
   DTS_SUBDOM = LEGISLATION AND FM ~("Regulation” OR "Directive" OR "Decision") AND AU_CODED = EP NOT FM_CODED = CORRIGENDUM AND DD >= 01/01/2000 <= 31/12/2019 AND (TE ~((distributed NEAR10 generation) OR (distributed NEAR10 solar) OR "net-metering" OR "feed-in-tariff"))

8. Batteries and storage
   DTS_SUBDOM = LEGISLATION AND FM ~("Regulation” OR "Directive" OR "Decision") AND AU_CODED = EP NOT FM_CODED = CORRIGENDUM AND DD >= 01/01/2000 <= 31/12/2019 AND (TE ~lithium-ion OR flywheel OR (electric NEAR10 charge) OR (charging NEAR10 station) OR (geothermal NEAR10 energy) OR (geothermal NEAR10 heating) OR (heat NEAR10 pumps) OR (direct NEAR10 air NEAR10 capture) OR (charging NEAR10 infrastructure) OR (electricity NEAR10 storage) OR (energy NEAR10 storage) OR (storage NEAR10 renewable) OR (storage NEAR10 renewable) OR (storage NEAR10 grid) OR (pumped NEAR10 hydro) OR (lead NEAR10 acid) OR (flow NEAR10 battery) OR (thermal NEAR10 storage) OR (battery NEAR10 storage) OR (heat NEAR10 storage) OR (cold NEAR10 storage) OR (pumped NEAR10 storage NEAR10 projects) OR battery)

9. Electric vehicles
   DTS_SUBDOM = LEGISLATION AND FM ~("Regulation” OR "Directive" OR "Decision") AND AU_CODED = EP NOT FM_CODED = CORRIGENDUM AND DD >= 01/01/2000 <= 31/12/2019 AND (TE ~ (electric NEAR10 vehicle) OR (electric NEAR10 bike) OR (electric NEAR10 bicycle) OR (electric NEAR10 scooter) OR (electric NEAR10 motorbike) OR (electric NEAR10 motorcycle) OR (electric NEAR10 car) OR (electric NEAR10 sedan) OR (electric NEAR10 SUV) OR (electric NEAR10 pick-up) OR (electric NEAR10 truck) OR (electric NEAR10 semi) OR (electric NEAR10 van) OR (electric NEAR10 bus) OR (electric NEAR10 autobus) OR (electric NEAR10 drive-train) OR (electric NEAR10 engine) OR (electrified NEAR10 vehicle) OR (electrified NEAR10 bike) OR (electrified NEAR10 bicycle) OR (electrified NEAR10 scooter) OR (electrified NEAR10 motorbike) OR (electrified NEAR10 motorcycle) OR (electrified NEAR10 car) OR (electrified NEAR10 sedan) OR (electrified NEAR10 SUV) OR (electrified NEAR10 pick-up) OR (electrified NEAR10 truck) OR (vehicle NEAR10 electrification) OR (vehicle-to-grid NEAR10 algorithms) OR (vehicle-to-grid NEAR10 storage) OR (grid to vehicle) OR (hybrid NEAR10 vehicle) OR (hybrid NEAR10 car) OR (hybrid NEAR10 SUV) OR (hybrid NEAR10 bus) OR (hybrid NEAR10 pick-up) OR (hybrid NEAR10 truck) OR (hybrid NEAR10 electric NEAR10 drive))

10. Biothermal bioenergy
    DTS_SUBDOM = LEGISLATION AND FM ~("Regulation” OR "Directive" OR "Decision") AND AU_CODED = EP NOT FM_CODED = CORRIGENDUM AND DD >= 01/01/2000 <= 31/12/2019 AND (TE ~(biofuel* OR bioenergy OR (cellulosic NEAR10 ethanol) OR (cellulosic NEAR10 technology) OR biogas OR biodiesel OR (energy NEAR10 crop) OR (anaerobic NEAR10 digester) OR (landfill NEAR10 gas) OR (wood NEAR10 waste) OR (agriculture NEAR10 waste) OR (agricultural NEAR10 waste) OR

(ethanol NEAR10 fuel) OR (ethanol NEAR10 gasolines) OR (corn NEAR10 ethanol) OR (sugar NEAR10 ethanol) OR (forest NEAR10 biomass) OR (cellulosic NEAR10 biomass) OR (waste NEAR10 biomass)))

11. Heating and cooling
DTS_SUBDOM = LEGISLATION AND FM ~("Regulation" OR "Directive" OR "Decision") AND AU_CODED = EP NOT FM_CODED = CORRIGENDUM AND DD >= 01/01/2000 <= 31/12/2019 AND (TE ~("Solar air condition" OR (waste NEAR10 heat) OR (heat NEAR10 recovery) OR (Ocean NEAR10 Thermal NEAR10 Energy NEAR10 Conversion) OR OTEC OR (combined NEAR10 heat NEAR10 power)))

Metadata included in the dataset

References
Title
ELI

Publication reference
Publication reference

Dates
Date of document
Date of publication
Date of effect
Date of end of validity

Classifications
EUROVOC descriptor
Subject matter

Miscellaneous information
Author
Form
Treaty
In force indicator
Instruments involved
Co-author

Relationship between documents
Legal basis
Amendment to
Amended by
Instruments cited
Transposed legal act(s)

Technical Data
Date created
Latest modification of notice
Cellar reference

II. List of legal texts

Labelled EU policies	
**CELEX number**	**Title**
32006L0066	Directive 2006/66/EC of the European Parliament and of the Council of 6 September 2006 on batteries and accumulators and waste batteries and accumulators and repealing Directive 91/157/EEC
32008R1099	Regulation (EC) No 1099/2008 of the European Parliament and of the Council of 22 October 2008 on energy statistics
32009L0028	Directive 2009/28/EC of the European Parliament and of the Council of 23 April 2009 on the promotion of the use of energy from renewable sources and amending and subsequently repealing Directives 2001/77/EC and 2003/30/EC
32009R0079	Regulation (EC) No 79/2009 of the European Parliament and of the Council of 14 January 2009 on type-approval of hydrogen-powered motor vehicles, and amending Directive 2007/46/EC
32012L0027	Directive 2012/27/EU of the European Parliament and of the Council of 25Â October 2012 on energy efficiency, amending Directives 2009/125/EC and 2010/30/EU and repealing Directives 2004/8/EC and 2006/32/EC
32013R0174	Regulation (EU) No 174/2013 of the European Parliament and of the Council of 5Â February 2013 amending Regulation (EC) NoÂ 106/2008 on a Community energy-efficiency labelling programme for office equipment
32013R0525	Regulation (EU) No 525/2013 of the European Parliament and of the Council of 21Â May 2013 on a mechanism for monitoring and reporting greenhouse gas emissions and for reporting other information at national and Union level relevant to climate change and repealing Decision No 280/2004/EC
32014R0421	Regulation (EU) No 421/2014 of the European Parliament and of the Council of 16 April 2014 amending Directive 2003/87/EC establishing a scheme for greenhouse gas emission allowance trading within the Community, in view of the implementation by 2020 of an international agreement applying a single global market-based measure to international aviation emissions
32018L2001	Directive (EU) 2018/2001 of the European Parliament and of the Council of 11 December 2018 on the promotion of the use of energy from renewable sources
32018R1999	Regulation (EU) 2018/1999 of the European Parliament and of the Council of 11 December 2018 on the Governance of the Energy Union and Climate Action, amending Regulations (EC) No 663/2009 and (EC) No 715/2009 of the European Parliament and of the Council, Directives 94/22/EC, 98/70/EC, 2009/31/EC, 2009/73/EC, 2010/31/EU, 2012/27/EU and 2013/30/EU of the European Parliament and of the Council, Council Directives 2009/119/EC and (EU) 2015/652 and repealing Regulation (EU) No 525/2013 of the European Parliament and of the Council

32019L0944	Directive (EU) 2019/944 of the European Parliament and of the Council of 5 June 2019 on common rules for the internal market for electricity and amending Directive 2012/27/EU
32019R0631	Regulation (EU) 2019/631 of the European Parliament and of the Council of 17 April 2019 setting CO2 emission performance standards for new passenger cars and for new light commercial vehicles, and repealing Regulations (EC) No 443/2009 and (EU) No 510/2011
32009R0397	Regulation (EC) No 397/2009 of the European Parliament and of the Council of 6 May 2009 amending Regulation (EC) No 1080/2006 on the European Regional Development Fund as regards the eligibility of energy efficiency and renewable energy investments in housing
32006L0032	Directive 2006/32/EC of the European Parliament and of the Council of 5 April 2006 on energy end-use efficiency and energy services and repealing Council Directive 93/76/EEC
32004L0008	Directive 2004/8/EC of the European Parliament and of the Council of 11 February 2004 on the promotion of cogeneration based on a useful heat demand in the internal energy market and amending Directive 92/42/EEC
32014L0094	Directive 2014/94/EU of the European Parliament and of the Council of 22 October 2014 on the deployment of alternative fuels infrastructure
32018L0844	Directive (EU) 2018/844 of the European Parliament and of the Council of 30 May 2018 amending Directive 2010/31/EU on the energy performance of buildings and Directive 2012/27/EU on energy efficiency
32014R0256	Regulation (EU) No 256/2014 of the European Parliament and of the Council of 26 February 2014 concerning the notification to the Commission of investment projects in energy infrastructure within the European Union, replacing Council Regulation (EU, Euratom) No 617/2010 and repealing Council Regulation (EC) No 736/96

III. Coding scheme

Reading instructions:

The coding scheme is ordered according to the hierarchical levels introduced in the main text - i.e., layer, feature, tag - and therefore is presented in this arrangement:
#. Layer [Layer name in data]
- Feature [Feature name in data]
  - Tag [Tag name in data]

1. Instrument types

- Instrument type [InstrumentType]
  - Voluntary agreement [VoluntaryAgrmt]: Refers to measures that are undertaken voluntarily by government agencies or industry bodies, based on a formalized agreement. There are incentives and benefits to undertaking the action, but generally few legal penalties in case of non-compliance. The scope of the action tends to be agreed upon in concert with the relevant actors; for example, agreements to report RD&D activities or non-binding commitments to cooperation between actors.
  - Framework policy [FrameworkPolicy]: Refers to the processes undertaken to develop and implement policies. This generally covers strategic policies that are intended to guide the development of further policies by, for example, defining long-term goals .
  - Tradable permit [TradablePermit]: Refers to GHG emissions trading schemes or white certificate systems related to energy efficiency or energy savings obligations. In the former, industries must hold permits to cover their GHG emissions; if they emit more than the amount of permits they hold, they must purchase permits to make up the shortfall, creating an incentive to reduce energy use.
  - Regulatory instrument [RegulatoryInstr]: Covers a wide range of instruments by which a government will oblige actors to undertake specific measures and/or report on specific information. Examples include

obligations on addressees to reduce energy consumption, mandatory standards, or requirements to report on GHG emissions or energy use.

- Tax incentives [TaxIncentives]: Policies to encourage or stimulate certain activities or behaviours through tax exemptions, tax reductions or tax credits on the purchase or installation of certain goods and services.
- Subsidies and direct incentives [Subsidies_Incentives]: Policies to stimulate certain activities, behaviours, or investments through subsidies or rebates for, e.g., the purchase of energy-efficient equipment or electric vehicles, grants, preferential loans and third-party financing for low-carbon technology manufacturing.
- Research, Development & Demonstration (RD&D) [RD_D]: Policies and measures for the government to invest directly in or facilitate investment in technology research, development, demonstration and deployment activities.
- Public Investment [PublicInvt]: Policies and measures guiding investment by public bodies. These include government procurement programs (e.g. the installation of solar PV on government buildings) and infrastructure investment (e.g. EV charging infrastructure).
- Education and outreach [Edu_Outreach]: Policies and measures designed to increase knowledge, awareness, and training among relevant stakeholders or users, including information campaigns, training programs, labelling schemes.
- Unspecified [Unspecified]: Expressions that describe policies and measures in general, or those that are not immediately attributable to a specific instrument type.

2. Policy design characteristics

- Time [Time]
  - Policy duration time [Time_PolDuration]: A set time frame that the policy is in place or a deadline by when it expires.
  - Monitoring time [Time_Monitoring]: Deadlines, time frames and frequencies related to monitoring.
  - Resources time [Time_Resources]: Temporal provisions around the resource allocation.
  - Compliance time [Time_Compliance]: The deadline or time frame for compliance with the regulation.
  - In-effect time [Time_InEffect]: The start date or effective date of the policy.

- Actor [Actor]
  - Default authority [Authority_default]: The individual or entity that is making the rule, ensuring its implementation, for enforcing the rule and may apply sanctioning, including an existing individual or entity empowered, directed or required to implement.
  - Legislative authority [Authority_legislative]: The individual or entity that is drafting or voting on legislation.
  - Newly established authority [Authority_established]: A newly established entity that is ensuring the policy's implementation.
  - Monitoring authority [Authority_monitoring]: An individual or entity responsible for monitoring the outcome of the policy, through report, review, or audit. All entities that are part of the monitoring process, and not the primary monitored entity.
  - Default addressee [Addressee_default]: The individual or entity that the rule applies to and needs to ensure its implementation.
  - Resources addressee [Addressee_resource]: The actor that receives a resource.
  - Monitored addressee [Addressee_monitored]: An individual or entity monitored for the outcome of the policy, through report, review, or audit.
  - Sector addressee [Addressee_sector]: Relevant sectors that are covered by the policy.

- Objective [Objective]
  - Quantitative target [Objective_QuantTarget]: A quantitative target or objective of the policy.
  - Qualitative intention [Objective_QualIntention]: A qualitatively stated intention or objective of the policy. This lacks a specific quantity that is targeted, for example increasing the amount of hydrogen produced with renewable electricity sources. Also includes references to unspecified targets.
  - Quantitative target not mitigation [Objective_QuantTarget_noCCM]: A quantitative target or objective of the policy, not pertaining to climate change mitigation (e.g. adaptation or employment targets).
  - Qualitative intention not mitigation [Objective_QualIntention_noCCM]: A qualitatively stated intention or objective of the policy, not pertaining to climate change mitigation (e.g. adaptation or employment targets). This lacks a specific quantity that is targeted.

- Resource [Resource]

    - Monetary spending [Resource_MonSpending]: Resources that are provided through government spending. Can be a concrete sum or unspecific assumption such as “more spending on…”. This includes grants, subsidies, allocations of funds.
    - Monetary revenues [Resource_MonRevenues]: Provisions that affect government revenue (positively or negatively). Can be a concrete sum or unspecific assumption such as “increase revenue”. This includes, e.g., tax credits (negative), tolls, fees, customs (positive).
    - Other resource type [Resource_Other]: Other resources such as personnel, facilities/equipment, or emissions allowances.

- Compliance [Compliance]
    - Sanctioning form [Form_sanctioning]: Sanctioning provisions and measures.
    - Monitoring form [Form_monitoring]: The form of the monitoring (provisions relating to report, review, or audit; standards and certification schemes)

- Reversibility [Reversibility]
    - Provision for reversibility [Reversibility_policy]: A provision for the extension or termination of the policy.

- Reference to other policies [Reference]
    - Reference to other policy [Ref_OtherPolicy]: External legislative text referenced for objectives, definitions, constraints, or for other reasons.
    - Amendment of policy [Ref_PolicyAmended]: Amendment of another policy, or repeal thereof, that is made through this legislation.
    - Reference to strategy or agreement [Ref_Strategy_Agreement]: Reference to treaties, constitutions, agreements, white papers, blue prints, overarching strategies. For example the Paris Agreement or key national climate strategies.

3. Technology and application specificity

- Technology specificity [TechnologySpecificity]
    - Low-carbon technology [Tech_LowCarbon]: Low-carbon technology, including renewable energy generation, storage, efficiency at various levels of precision.
    - Other technology [Tech_Other]: Other technologies with no direct role for decarbonization.
- Energy source/carrier specificity [EnergySpecificity]

    - Low-carbon energy source or carrier [`Energy_LowCarbon`]: A low-carbon energy source or energy carrier (includes biomass and nuclear).
    - Other energy source or carrier [`Energy_Other`]: Other energy source or energy carrier (includes fossil fuels).
- Application specificity [`ApplicationSpecificity`]
    - Low-carbon application [`App_LowCarbon`]: Application of a low-carbon technology or low-carbon application of a technology.
    - Other application [`App_Other`]: Other application with no direct role for decarbonization.

IV. Rule book

General rules:

- Knowledge from other parts of the text (not within that section) should not be used for making a coding decision; only information in the immediate text surrounding the passage in question.
- We are interested in information that can be extracted from the text, not in the structure of the text itself. For example, we want to know the number of a directive, not all the places where it is referenced. If that directive is referred to later in the text without providing any other name or number to specify it (e.g., by "the directive"), no need to code that.
- Scope of text to code:
  - Text quoted for an amendment should be coded.
  - We also annotate headings if from the language the necessary information becomes clear.
  - Independent of the topic, all text is relevant, e.g., we also code non-energy text parts. We do not annotate the preamble.
- Length of the span that is highlighted:
  - The length of a span can be chosen freely (unitization).
  - The span should be as short as possible without loss of primary information.
  - Sometimes longer spans can be subdivided into different (potentially overlapping) spans.
  - Avoid including articles such as "the" or "a" (unless in the middle of a span).
- Tenses:
  - We annotate irrespective of the tense.
- Two adjacent spans with the same label should in general be annotated separately, e.g., "**Directives 70/156/EEC** `[Ref_OtherPolicy]` and **80/1268/EEC** `[Ref_OtherPolicy]`". However, where terms occur frequently together, those also may be annotated as one (e.g., "batteries and accumulators" `[Tech_LowCarbon]`).
- Some spans may be annotated by several different tags from different features or layers. For example, annotating instrument types and policy design characteristics may be appropriate for the same span, e.g.

“national inventory system” can be `RegulatoryInstr` and `Form_monitoring`.

- ○ For the curation, we use all correct overlapping answers.

Rules specific to parts of the coding scheme:

Instrument types:

- Tags:
  - Voluntary agreement [`VoluntaryAgrmt`]: Measures that are undertaken voluntarily by governments or government agencies
  - Framework policy [`FrameworkPolicy`]: Strategic policies that are intended to guide the policy development of further policies by, for example, defining long-term goals.
  - Tradable permit [`TradablePermit`]: Refers to GHG emissions trading schemes or white certificate systems related to energy efficiency or energy savings obligations.
  - Regulatory instrument [`RegulatoryInstr`]: Wide range of instruments by which a government will oblige actors to undertake specific measures.
  - Tax incentives [`TaxIncentives`]: Policies to encourage or stimulate certain activities or behaviours through tax exemptions, tax reductions or tax credits on the purchase or installation of certain goods and services.
  - Subsidies and direct incentives [`Subsidies_Incentives`]: Policies to stimulate certain activities, behaviours, or investments through subsidies or rebates, grants, or preferential loans.
  - Research, Development & Demonstration (RD&D) [`RD_D`]: Policies and measures for the government to invest directly in or facilitate investment in technology research, development, demonstration and deployment activities.
  - Public Investment [`PublicInvt`]: Policies and measures guiding investment by public bodies.
  - Education and outreach [`Edu_Outreach`]: Policies and measures designed to increase knowledge, awareness, and training among relevant stakeholders or users.
  - Unspecified [`Unspecified`]: Expressions that describe policies and measures in general, or those that are not immediately attributable to a specific instrument type.

General:

- Feed-in tariffs are both `RegulatoryInstr` and `Subsidies_Incentives`.

- Auctions or auctioning mechanisms in themselves are components of instrument types (for example auctioning of permits), if they are kept general they fall under regulations.
- Intergovernmental agreements, such as the Paris Agreement, are not instrument types as such but are viewed as `Ref_Strategy_Agreement;` also, references to the European Energy Union should be labelled as `Ref_Strategy_Agreement.`
- Some expressions refer to technicalities of implementation and these are not annotated as instrument types. For example, in the text “Member states shall ensure a level playing field”, the expression “level playing field” is not annotated because it is a technicality of implementation.

Specific:

- Voluntary agreement `[VoluntaryAgrmt]`
    - Includes voluntary standards and certification schemes.
    - Intergovernmental agreements are not included here.
- Tradable permit `[TradablePermit]`
    - Should be used to identify descriptions of markets and mechanisms that use tradable permits. We do not label terms that describe the permits themselves as `TradablePermit:` “Emission credits”, e.g., are `Resource_other`.
    - “Energy Performance Contracting (EPC)” and “electricity market” are also examples of `TradablePermit.`
- Regulatory instrument `[RegulatoryInstr]`
    - Self-referential, repeated descriptions alone like “this Regulation” should not be labelled. What follows these self-referential descriptions, however, should be labelled: “This Regulation establishes a **mechanism** `[Unspecified]` for…”
    - Mandatory standards and certification schemes are included.
    - Verbs on their own such as “prohibit”, “require”, etc. should not be labelled as `RegulatoryInstr.`
    - “Rules and regulations” are `RegulatoryInstr` (unless the term “rules” is used only very broadly).
- Tax incentives [`TaxIncentives`]
    - Tariffs in general are `TaxIncentives.`
- Subsidies and direct incentives [`Subsidies_Incentives`]
    - Also includes “direct price support schemes”
- Public Investment `[PublicInvt]`
    - Includes “international development” action if this refers to an investment.
- Education and outreach `[Edu_Outreach]`
    - Includes labelling schemes to inform or educate.

- Unspecified [Unspecified]
    - Expressions that are not further specified like “support scheme”, “implementing measures” and “market based measures” should be labelled as Unspecified.

Policy design characteristics:

- Time:
    - Tags:
        - Policy duration time [Time_PolDuration]: A set time frame that the policy is in place or a deadline by when it expires.
        - Monitoring time [Time_Monitoring]: Deadlines, time frames and frequencies related to monitoring.
        - Resources time [Time_Resources]: Temporal provisions around the resource allocation.
        - Compliance time [Time_Compliance]: The deadline or time frame for compliance with the regulation.
        - In-effect time [Time_InEffect]: The start date or effective date of the policy.

    - Rules:
        - Mainly, we are interested in identifying statements about a policy’s start and end date. Dates that relate to when a policy is approved or adopted are not relevant in this sense.
            - E.g., do not label: "Done at Brussels, 18 June 2020."
        - We consider a policy being in force as long as it is not explicitly repealed, revoked, or amended.
        - Compliance [Time_Compliance] vs policy duration time [Time_PolDuration]: When addressees are required to do something in a given time frame, this should be labelled as Time_Compliance. As in the following example, this might be described as a certain timeframe within the duration of the overarching policy:
            - “The manufacturer shall ensure that its average specific emissions of CO2 do not exceed the following specific emission standards: (a) **for the calendar year 2020** [Time_Compliance], the specific emissions…” The time indicated here does not pertain to the duration of the policy as such but to the time frame during which the addressees have to do something.

        - The tag `Time_Resources` is to be used to distinguish between resources themselves and the time dimension:
            - "There are authorised to be appropriated, for activities under this section, a total of **EUR 50,000,000** [`Resource_MonSpending`] for fiscal years 1997 and 1998 [`Time_Resources`]..."
        - Preferably, only code expressions for time itself, not prepositions such as “by”, “on”, “after” etc.
            - "The Commission shall assess that obligation, with a view to submitting, by **2023** [`Time_Compliance`], a legislative proposal ..."
            - “By **25 June 2019** [`Time_Monitoring`] and **every two years thereafter** [`Time_Monitoring`], the Commission shall review the list of feedstock set out in Parts A and B of Annex IX…”
        - The tag `Time_InEffect` is to be used to identify the date from which a policy is in effect, not the date a policy was passed or adopted.

- Actor:
    - Tags:
        - Default authority [`Authority_default`]: The individual or entity that is making the rule, ensuring its implementation, for enforcing the rule and may apply sanctioning, including an existing individual or entity empowered, directed or required to implement.
        - Legislative authority [`Authority_legislative`]: The individual or entity that is drafting or voting on legislation.
        - Newly established authority [`Authority_established`]: A newly established entity that is ensuring the policy’s implementation.
        - Monitoring authority [`Authority_monitoring`]: An individual or entity responsible for monitoring the outcome of the policy, through report, review, or audit. All entities that are part of the monitoring process, and not the primary monitored entity.
        - Default addressee [`Addressee_default`]: The individual or entity that the rule applies to and needs to ensure its implementation.
        - Resources addressee [`Addressee_resource`]: The actor that receives a resource.
        - Monitored addressee [`Addressee_monitored`]: An individual or entity monitored for the outcome of the policy, through report, review, or audit.
        - Sector addressee [`Addressee_sector`]: Relevant sectors that are covered by the policy.

- Rules:
    - Regarding monitoring, the distinction between actors *doing* the monitoring and those *being* monitored is of most interest to us.
    - Actors that are actively involved in monitoring processes in the sense of conducting monitoring are labelled as `Authority_monitoring`. Also actors that utilise information gathered by someone else, for example for drafting a report based on reviews conducted by other actors, should be labelled as `Authority_monitoring`.
    - When actors are exempt from requirements in a regulation, for example when a regulation does not apply to a particular group (e.g., a sector), they should still be labelled as addressees (`Addressee_sector` in the example above).
    - All actors should be labelled individually so as to capture their unique roles. This also applies to lists of actors, irrespective of whether the same or different tags are used, for example as in "the European Commission [`Addressee_default`], the European Parliament [`Addressee_default`] and the Council [`Addressee_default`]…"
    - In EU policies, "Member states" are most often addressees (in the form of `Addressee_default`, `Addressee_resource` or `Addressee_monitored`).
    - Label "European Parliament" and "Council" as `Authority_legislative` when they are mentioned in relation to creating an act.
    - A policy can stipulate the creation of a new actor with authority over implementing. This should be labelled as in the following example:
        - "The **Commission** [`Authority_default`] shall be assisted by the **Technical Committee — Motor Vehicles (TCMV)** [`Authority_established`] established by Article 40(1) of Directive 2007/46/EC [`Ref_OtherPolicy`]."
    - Sectors are descriptions of groups of individuals and organisations that are concerned with certain economic activities. They can be described at varying levels of abstraction from broad (e.g., "private" sector), to more detailed (e.g., "industrial", "residential", or "transportation" sectors), to very detailed (e.g., "utilities" or"electricity undertakings").
        - For example: "In performing the task referred to in point (i) of paragraph 1, **transmission system operators**

[Addressee_sector] shall procure balancing services subject to the following: (a) transparent, non-discriminatory and market-based procedures; (b) the participation of all qualified **electricity undertakings** [Addressee_sector] and **market participants** [Addressee_sector], including **market participants offering energy from renewable sources** [Addressee_sector], **market participants engaged in demand response** [Addressee_sector], **operators of energy storage facilities** [Addressee_sector] and **market participants engaged in aggregation** [Addressee_sector]."

- In some instances, the same expression can describe both actors in a more general way or describe the sector as such. It is therefore not always warranted to label a group as Addressee_sector if actions are described that are irrelevant to their role as a sector.
- In cases where actors are simply mentioned in passing with no relevance to the policy at hand (for example mentioned for purposes of reference as in the example below), these actors need not to be labelled.
  - "The **Commission** [Authority_default] shall adopt implementing acts to set out rules on the structure, format and submission process of the information relating to national inventory systems (...) in accordance with relevant decisions adopted by the bodies of the UNFCCC or the Kyoto Protocol or of agreements deriving from them or succeeding them."

- Objectives:
  - Tags:
    - Quantitative target [Objective_QuantTarget]: A quantitative target or objective of the policy.
    - Qualitative intention [Objective_QualIntention]: A qualitatively stated intention or objective of the policy.
    - Quantitative target not mitigation [Objective_QuantTarget_noCCM]: A quantitative target or objective of the policy, not pertaining to climate change mitigation (e.g., jobs).
    - Qualitative intention not mitigation [Objective_QualIntention_noCCM]: A qualitatively stated intention or objective of the policy, not pertaining to climate change mitigation (e.g., jobs).

- Rules:
  - Primarily, we are interested in "material" goals related to climate change mitigation. These can be expressed in either qualitative ("mitigate climate change") or quantitative terms ("50% reduction of greenhouse gas emissions until 2030"). The latter can be very technical or detailed (e.g. "increase energy efficiency in federal agency's use of photocopiers by 3.4% until 2021") but should still be included.
  - Objectives need to be distinguished from the means how to achieve them, i.e. policy instrument types. In the example below, the (qualitative) objective is described in the second part of the sentence ("achieve the…") whereas the first part of the sentence ("the hydrogen…") describes how to achieve this objective:
    - "To authorise the **hydrogen** [`Tech_LowCarbon`] **research, development, and demonstration programs** [`RD_D`]… to **achieve the more economic hydrogen production and use** [`Objective_QualIntention`]".
  - For `Objective_QuantTarget`, we are interested in substantive information pertaining to the target, such as its coverage (e.g., greenhouse gases, sectors), the reductions quantified and the related baseline, and its time frame. Additional information, e.g., linking to other parts of a policy should not be included when labelling.
    - For example: "Each Member State shall, in 2030, **limit its greenhouse gas emissions at least by the percentage set for that Member State in Annex I in relation to its greenhouse gas emissions in 2005** [`Objective_QuantTarget`], determined pursuant to paragraph 3 of this Article."
  - If a quantifiable goal is not explicitly stated but could be calculated or is further specified in another part of the policy, it should also be labelled as `Objective_QuantTarget`. For example, "...the **specific emissions target** [`Objective_QuantTarget`] determined in accordance with points 1 and 2 of Part A of Annex I..."
  - Implicit mention of goals should sometimes be labelled if mentioned like a goal (e.g., "energy savings achieved"), but not if it is not mentioned as such (e.g. "... can produce energy savings")
  - Regarding qualitative intentions, also references to broader EU objectives should be included, for example: "The **objectives, targets and contributions** [`Objective_QualIntention`] as sufficient

for the collective achievement of the **Energy Union objectives** [Objective_QualIntention]”

- Resources:
  - Tags:
    - Monetary spending [Resource_MonSpending]: Resources that are provided through government spending, either in the form of direct allocation of funds or, e.g., in the form of grants and subsidies. Can be a concrete sum or unspecific assumption such as “more spending on…”.
    - Monetary revenues [Resource_MonRevenues]: Provisions that affect revenue (positively or negatively). Can be a concrete sum or unspecific assumption such as “increase revenue”. This includes, e.g., tax credits (negative), tolls, fees, customs (positive).
    - Other resource type [Resource_other]: Other resources, such as personnel, facilities/equipment, or emissions allowances.

  - Rules:
    - We are interested in the commitments made in terms of monetary or other resources. Regarding the former, the actual sum (e.g. “EUR50,000,000”) should be labelled.
    - Even miniscule sums, such as travel expenses, should be labelled Resource_MonSpending if they are specified in the text.
    - Resource_other can take various forms, as shown in the following examples:
      - “...may have a limited cancellation of up to a maximum of 100 million EU ETS allowances [Resource_other] collectively taken into account for their compliance under this Regulation.” Similarly, “annual emissions allocations” should be labelled as [Resource_other].
      - “...may appoint for a limited term, or on a temporary basis, scientists, engineers, and other technical and professional personnel [Resource_other] on leave of absence from academic, industrial, or research institutions to work for….”
    - Make sure to include all the necessary information related to resources, for example also percentages as in “**10% of the annual emissions allocations** [Resource_other]”.
    - General formulations like “financial support” and “support” should also be labelled as Resource_MonSpending or Resource_MonRevenues and Resource_other, respectively.

        - “Financial incentives” or “grants” as such would be an instrument type. If a resource is mentioned this should be labelled independently, for example: “...**grant** [`Subsidies_Incentives`] of **EUR10 million** [`Resource_MonSpending`]...” In some instances, the same span may be labelled as instrument type and resource.
        - If a policy’s addressees are required to invest their resources, i.e., resources that are neither revenues nor spending for the government or public actors, this should be labelled as `Resource_other`. Example: “**Member States** [`Authority_default`] shall oblige **producers, or third parties acting on their behalf** [`Addressee_sector`], to finance any **net costs** [`Resource_other`] arising from public information campaigns [`Edu_Outreach`].”
        - Data and (statistical) information can, under certain circumstances, also be understood as `Resource_other`, namely if they are made available to an actor for example for their economic advantage. In most cases, however, data should not be labelled in this way.

- Compliance:
    - Tags:
        - Sanctioning form [`Form_sanctioning`]: Sanctioning provisions and measures.
        - Monitoring form [`Form_monitoring`]: The form of the monitoring (provisions relating to report, review, or audit; standards and certification schemes)
    - Rules:
        - Regarding monitoring, only label its form, such as “report” etc., not further specifications of its content but include specifications such as “yearly”, ”biannual” etc.
            - For example: “a detailed **yearly report** [`Form_monitoring`] on the usage of pencils…”
        - How monitoring is conducted, i.e., `Form_monitoring`, can be described both through verbs and nouns. Also, monitoring can be “forward-looking” in the sense of using projections or modelling.
            - For example: “...**monitoring, reporting, reviewing and verifying** [`Form_monitoring`] greenhouse gas emissions and other information [`Form_monitoring`] pursuant to Article 6 of Decision No 406/2009/EC…” or equivalently: “...**monitoring** [`Form_monitoring`], **reporting** [`Form_monitoring`], **reviewing** [`Form_monitoring`] and **verifying** [`Form_monitoring`] greenhouse gas emissions and other information

        [Form_monitoring] pursuant to Article 6 of Decision No 406/2009/EC…"
        - "Member States shall make available to the public, in electronic form, their **national projections of greenhouse gas emissions by sources and removals by sinks** [Form_monitoring] along with relevant **technical reports** [Form_monitoring] that underpin those **projections** [Form_monitoring]...."

- Reversibility:
    - Tags:
        - Pol_duration_provision [Reversibility_policy]: A provision for the extension or termination of the policy.

    - Rules:
        - We are interested in expressions that describe processes of how (or when) a policy can be changed in the future.
            - For example: "The Commission [Authority_default] may, if the Member state [Addressee_default] has not met the indicative trajectory by a limited margin, and taking due account of the current and future measures [Unspecified] taken by the Member state [Addressee_default], **adopt a decision to release the Member state from the obligation to submit an amended national renewable energy action plan** [Reversibility_policy]." The final span would contain additional overlapping annotations ("...**Member state** [Addressee_default]..."; "...**national renewable energy action plan** [Ref_Strategy_Agreement]...").

- Links to other policies:
    - Tags:
        - Reference to other policy [Ref_OtherPolicy]: External legislative text referenced for objectives, definitions, constraints, or for other reasons. No amendments.
        - Amendment of policy [Ref_PolicyAmended]: Amendment of another policy or repeal thereof that is made through this legislation.
        - Reference to strategy or agreement [Ref_Strategy_Agreement]: Reference to treaties, constitutions, agreements, white papers, overarching strategies. For example, the Paris Agreement.
    - Rules:

- Often, other policies are mentioned to clarify or define matters. Such “technical” references should be labelled as Ref_OtherPolicy. In general, highlighting should be as concise as possible, e.g. by only highlighting a reference’s main identifier as in “Article 7 of **Decision No 406/2009/EC** [Ref_OtherPolicy]”.
- We are also interested in expressions that detail how another policy is amended or changed. Note that another policy can also be (partly) repealed through the text at hand. Such expressions should be labelled as Ref_PolicyAmended as in “**Regulation (EU) 2019/2088** [Ref_PolicyAmended] should further be amended to…”.
- In text segments amending other policies, references to yet other policies should be labelled as well.
- When it is not perfectly clear from the text that a mentioned policy is being amended, it should be labelled as Ref_OtherPolicy.
- Be aware that the same policy mentioned in a text can fulfil different roles in various contexts: it can be referenced for clarification and thus be Ref_OtherPolicy in one context while being amended and thus be Ref_PolicyAmended in another.
- In general, only annotate the name of the policy (not “Article” or “Section”). For example “Decision No 406/2009/EC” (instead of “Article 7 of Decision No 406/2009/EC”).
- If a policy is referenced by a general name (e.g., “the Directive”) after being mentioned before, only highlight the part where the policy is explicitly named.
- The IPCC and its reports do not need to be labelled.
- The following example should be labelled as Ref_other_policy, and not Policy_amended, since it is mentioned in passing that the policy is also amended:“if the CO2 emission and fuel consumption figures have been determined in accordance with the requirements of **Directive 80/1268/EEC** [Ref_other_policy], as amended by this Directive”.
- Targets in themselves are not Ref_Strategy_Agreement, they are not strategies.
- Occasionally, formulations like “without prejudice to Union and national laws” are used. This should be labelled as Ref_OtherPolicy as the expression points to the whole corpus of Union and Member state law(s) pertinent to this specific circumstance. For example, (the fundamental principles of) EU competition law are seen as being of overriding importance.-

        - When the expression “internal energy market” is used, it should be labelled as `Ref_Strategy_Agreement` as this expression refers to an element of the EU’s common market that is codified in the Treaty on the Functioning of the European Union. Similarly, references to the “(European) Energy Union” should also be labelled as `Ref_Strategy_Agreement`.

3. Technology and application specificity:

- Rules:
    - We are interested in identifying expressions of low carbon technologies, energy carriers, and applications. Generally, this includes:
        - Renewable energy technologies
        - Hydrogen technologies
        - Energy efficiency measures
        - Electrification approaches (including digital technologies that enable that)
        - Energy storage technologies
        - Biomass-based fuels
        - Nuclear
    - Fossil fuels (including natural gas) are not considered.

- Technology specificity [`TechnologySpecificity`]
    - Tags:
        - Low-carbon technology [`Tech_LowCarbon`]: Low-carbon technology, including renewable energy generation, storage, efficiency at various levels of precision, carbon capture technology.
        - Other technology [`Tech_Other`]: Other technologies with no direct role for decarbonization.
    - Rules:
        - Compound words that include technology words (e.g. in “aircraft operator”) are not to be labelled as `Tech_Other`.
        - Enabling or auxiliary technologies such as energy efficiency tools etc. are included in `Tech_LowCarbon`. Similarly, expressions that mention electricity transmission and distribution systems as a means to integrate more renewables, should be labelled as `Tech_LowCarbon`. However, if transmission and distribution systems are discussed only in general terms (e.g., as in “secure, reliable, and

efficient transmission system"), these should be labelled as Tech_Other.

    - Energy-efficient buildings and building renovations or retrofits should be labelled Tech_LowCarbon, particularly when expressions like "deep (energy) renovation," "zero carbon buildings," or "passive houses" etc. are used.
    - Expressions describing (light and/or heavy-duty) vehicles as such should be labelled Tech_Other (as, e.g., in "This Regulation establishes CO2 emissions performance requirements for new **passenger cars** [Tech_Other] and for new **light commercial vehicles** [Tech_Other]..."). Only expressions clearly describing electric vehicles should be labelled as Tech_LowCarbon (as, e.g., in "foster **electric vehicle** [Tech_LowCarbon] deployment"). If the focus is more specifically on electrification of vehicles by the use of batteries, this should be labelled as App_LowCarbon.
    - Hydrogen should only be labelled if it is part of an application or technology description.
    - The "electricity generation" can, in some contexts, also be used to describe a sector being targeted. In these cases, the expression should be labelled as Addressee_sector and not Tech_Other.

- Energy source/carrier specificity [EnergySpecificity]
  - Tags:
    - Low-carbon energy source or carrier [Energy_LowCarbon]: A low-carbon energy source or energy carrier (includes biomass and nuclear).
    - Other energy source or carrier [Energy_Other]: Other energy source or energy carrier (includes fossil fuels).
  - Rules
    - Electricity is an energy carrier.
    - Energy source or carrier expressions (such as "electricity") that appear in compound words relating to technologies (or applications) should not be labelled separately. E.g., "electricity infrastructure" should only be labelled as Tech_LowCarbon.
    - Whether an energy source or carrier is low carbon can also depend on how it is created, which may be described in the context. For example: "...where **electricity** [Energy_LowCarbon] is used for the production of **renewable liquid** [Energy_LowCarbon] and **gaseous transport fuels of non-biological origin** [Energy_LowCarbon],

either directly or for the production of **intermediate products** [Energy_LowCarbon]...”

- Application specificity [ApplicationSpecificity]
  - Tags:
    - Low-carbon application [App_LowCarbon]: Application of a low-carbon technology or low-carbon application of a technology.
    - Other application [App_Other]: Other application with no direct role for decarbonization.
  - Rules:
    - Expressions of “demand response” and “energy storage” should be labelled as App_LowCarbon. However, expressions that explicitly (or clearly from the context) aim at technologies for energy storage (e.g., “energy storage technology”), should be labelled as Tech_LowCarbon.
    - If an application is connected to a low-carbon technology, then the related expression should be labelled as App_LowCarbon.
    - Regarding batteries and vehicles, expressions like “batteries for electric vehicles” should be labelled as App_LowCarbon because the focus here is on electrification (and thus decarbonization) via batteries. If expressions refer to other applications for batteries that are not for the purpose of electrification/decarbonization (e.g. igniting a car or in military appliances), these should be labelled App_Other.
    - “Energy services”, “balancing of the electricity grid” and “ancillary services” should be labelled as App_Other.

Datasheet for dataset POLIANNA

S. Sewerin, L. H. Kaack, J. Küttel, F. Sigurdsson,
O. Martikainen, A. Esshaki, and F. Hafner

November 27, 2023

This is a datasheet for the dataset POLIcy design ANNotAtions (POLIANNA) based on the questions developed by Gebru et al. [2021]. Many aspects regarding the data are described in the main text of the accompanying paper entitled "Towards understanding policy design through text-as-data approaches: The polcy design annotations (POLIANNA) dataset". Here, we keep answers short in order to reduce redundancy.

1 Motivation

- **For what purpose was the dataset created? Was there a specific task in mind? Was there a specific gap that needed to be filled?** See the accompanying paper. For reference, the abstract reads: "Despite the importance of ambitious policy action for addressing climate change, large and systematic assessments of public policies and their design are lacking as analysing text manually is labour-intensive and costly. POLIANNA is a dataset of policy texts from the European Union (EU) that are annotated based on theoretical concepts of policy design, which can be used to develop supervised machine learning approaches for scaling policy analysis. The dataset consists of 20,577 annotated spans, drawn from 18 EU climate change mitigation and renewable energy policies. We developed a novel coding scheme translating existing taxonomies of policy design elements to a method for annotating text spans that consist of one or several words. Here, we provide the coding scheme, a description of the annotated corpus, and an analysis of inter-annotator agreement, and discuss potential applications. As understanding policy texts is still difficult for current text-processing algorithms, we envision this database to be used for building tools that help with manual coding of policy texts by automatically proposing paragraphs containing relevant information."
- **Who created the dataset (e.g., which team, research group) and on behalf of which entity (e.g., company, institution, organization)?** The dataset is created by the authors of the accompanying paper listed on top of this datasheet. The work was not done on behalf of any entity.

- **Who funded the creation of the dataset? If there is an associated grant, please provide the name of the grantor and the grant name and number.** It was supported by the Swiss National Science Foundation (grant number CRSK-1_190936) and a ETH Career Seed Grant SEED-24 19-2, funded by the ETH Zurich Foundation.

2 Composition

- **What do the instances that comprise the dataset represent? Are there multiple types of instances?** The dataset consists of annotated text divided into spans by the annotators (unitizing). Every span corresponds to one instance, which is annotated with three labels in a hierarchical fashion. The lowest level label is a "tag", which belongs to a "feature", and which in turn belongs to a "layer." We organize the dataset by "articles" that correspond to one article in a legislative text. Those splits have no other meaning but to structure the data.

- **How many instances are there in total (of each type, if appropriate)?** There are in total 20577 annotated spans. A summary overview can be found in the paper and can be computed with notebooks available in the online repository at `https://github.com/kueddelmaier/POLIANNA`.

- **Does the dataset contain all possible instances or is it a sample (not necessarily random) of instances from a larger set? If the dataset is a sample, then what is the larger set? Is the sample representative of the larger set (e.g., geographic coverage)? If so, please describe how this representativeness was validated/verified. If it is not representative of the larger set, please describe why not (e.g., to cover a more diverse range of instances, because instances were withheld or unavailable).** The dataset is a subset of all legal acts available by the European Union (i.e., regulations, directives, decisions, recommendations and opinions) at Publications Office of the European Union [2022]. Please refer to Section "Usage notes" of the paper for case selection.

- **What data does each instance consist of? "Raw" data (e.g., unprocessed text or images) or features?** Each instance consists of of a segment of raw text with minimal pre-processing that was segmented manually.

- **Is there a label or target associated with each instance?** There is a label associated with each instance, and and the same span may occur several times with a different label. See above and the paper for a detailed description of the labels.

- **Is any information missing from individual instances?** There is no information missing from individual instances.

- **Are relationships between individual instances made explicit?** Individual instances may overlap in the text that is included in the span, as such overlap was explicitly allowed during annotation.

- **Are there recommended data splits (e.g., training, development, validation, testing)?** We recommend to stratify by legislative articles to avoid overlapping spans to leak between splits.

- **Are there any errors, sources of noise, or redundancies in the dataset? If so, please provide a description.** The dataset is a curated version based on annotations by two annotators. We extensively evaluated inter-annotator agreement in the main paper. In addition, policy text is at times repetitive. As such there are parts of sentences in different text passages that are exactly the same, and therefore the dataset can contain redundant annotations, which belong to different text but read the same.

- **Is the dataset self-contained, or does it link to or otherwise rely on external resources?** The dataset is self-contained.

- **Does the dataset contain data that might be considered confidential (e.g., data that is protected by legal privilege or by doctor–patient confidentiality, data that includes the content of individuals' non-public communications)? Does the dataset contain data that, if viewed directly, might be offensive, insulting, threatening, or might otherwise cause anxiety?** The data and labels do not contain confidential or offensive data.

3 Collection process

- **How was the data associated with each instance acquired? Was the data directly observable, reported by subjects, or indirectly inferred/derived from other data?** The raw data was directly acquired, and labels where created based on the description in the paper.

- **What mechanisms or procedures were used to collect the data (e.g., hardware apparatuses or sensors, manual human curation, software programs, software APIs)? How were these mechanisms or procedures validated?** The data were directly downloaded from the EUR-Lex website Publications Office of the European Union [2022] using their download functionality.

- **If the dataset is a sample from a larger set, what was the sampling strategy (e.g., deterministic, probabilistic with specific sampling probabilities)?** The dataset is a subset of all available European laws. Please see Section "Policy selection" in the paper for a description of our sample selection. Policies were selected deterministically based on certain criteria.

- **Who was involved in the data collection process and how were they compensated?** The data collection process only involved student assistants at ETH Zürich who received hourly rates according to university policy.

- **Over what timeframe was the data collected? Does this timeframe match the creation timeframe of the data associated with the instances (e.g., recent crawl of old news articles)? If not, please describe the timeframe in which the data associated with the instances was created.**

- **Were any ethical review processes conducted?** This was not deemed necessary.

4 Preprocessing/cleaning/labeling

- **Was any preprocessing/cleaning/labeling of the data done (e.g., discretization or bucketing, tokenization, part-of-speech tagging, SIFT feature extraction, removal of instances, processing of missing values)? If so, please provide a description.** Please see the paper.

- **Was the "raw" data saved in addition to the preprocessed/cleaned/labeled data (e.g., to support unanticipated future uses)? If so, please provide a link or other access point to the "raw" data.** The raw data can be accessed at Publications Office of the European Union [2022].

- **Is the software that was used to preprocess/clean/label the data available? If so, please provide a link or other access point.** We used own scripts that are available at `https://github.com/kueddelmaier/POLIANNA` and an open source labeling tool provided by TU Darmstadt, Inception Klie et al. [2018].

5 Uses

- **Has the dataset been used for any tasks already? If so, please provide a description.** The dataset has been used in a PhD thesis in the summer of 2023 which is available here `https://doi.org/10.3929/ethz-b-000641426`.

- **What (other) tasks could the dataset be used for?** Please refer to Section "Usage notes" of the paper.

- **Is there anything about the composition of the dataset or the way it was collected and preprocessed/cleaned/labeled that might impact future uses? For example, is there anything that a dataset consumer might need to know to avoid uses that could result in unfair treatment of individuals or groups (e.g., stereotyping,**

quality of service issues) or other risks or harms (e.g., legal risks, financial harms)? Is there anything a dataset consumer could do to mitigate these risks or harms? Some parts of the dataset pertain only to climate change mitigation and renewable energy, and given the selection of documents included in the dataset, they may not cover the entire possible instances of general policy-making. This means that the dataset may need to be expanded for other use cases in the future. We intended to provide the necessary scripts and descriptions for users to expand the dataset but cannot guarantee that the tools and sources remain directly compatible with those. Users should be able to, however, recreate the annotations also with other tools.

- **Are there tasks for which the dataset should not be used?** While there are many tasks that the dataset is not appropriate for, there are no known use cases that may lead to intentionally or unintentionally malicious use.

6 Distribution

- **Will the dataset be distributed to third parties outside of the entity on behalf of which the dataset was created?** The dataset is publicly available with a digital object identifier (DOI) at `https://doi.org/10.5281/zenodo.7569275`.

7 Maintenance

- **Who will be supporting/hosting/maintaining the dataset?** The dataset will be supported by the authors of the accompanying paper.

- **How can the owner/curator/manager of the dataset be contacted (e.g., email address)?** The authors can be contacted via their institutional email addresses.

- **Will the dataset be updated (e.g., to correct labeling errors, add new instances, delete instances)? If so, please describe how often, by whom, and how updates will be communicated to dataset consumers (e.g., mailing list, GitHub)?** There is currently no plan to update the dataset. If updates are made, they will be made as new versions on the data repository.

- **If others want to extend/augment/build on/contribute to the dataset, is there a mechanism for them to do so? If so, please provide a description. Will these contributions be validated/verified?** There is currently no plan to have others contribute to updates of the datasets. If this changes in the future it will be communicated at `https://github.com/kueddelmaier/POLIANNA`.

References

Timnit Gebru, Jamie Morgenstern, Briana Vecchione, Jennifer Wortman Vaughan, Hanna Wallach, Hal Daumé Iii, and Kate Crawford. Datasheets for datasets. *Communications of the ACM*, 64(12):86–92, 2021.

Publications Office of the European Union. EUR-Lex: Access to European Union law, 2022. Available at `https://eur-lex.europa.eu/homepage.html`.

Jan-Christoph Klie, Michael Bugert, Beto Boullosa, Richard Eckart de Castilho, and Iryna Gurevych. The INCEpTION Platform: Machine-Assisted and Knowledge-Oriented Interactive Annotation. In *Proceedings of the 27th International Conference on Computational Linguistics: System Demonstrations*, pages 5–9. Association for Computational Linguistics, Juni 2018. URL `http://tubiblio.ulb.tu-darmstadt.de/106270/`. The 27th International Conference on Computational Linguistics (COLING 2018).
